# Rapid Desynchronization of an Electrically Coupled Interneuron Network with Sparse Excitatory Synaptic Input

**DOI:** 10.1016/j.neuron.2010.06.028

**Published:** 2010-08-12

**Authors:** Koen Vervaeke, Andrea Lőrincz, Padraig Gleeson, Matteo Farinella, Zoltan Nusser, R. Angus Silver

**Affiliations:** 1 Department of Neuroscience, Physiology and Pharmacology, University College London, London WC1E 6BT, UK; 2 Laboratory of Cellular Neurophysiology, Institute of Experimental Medicine of the Hungarian Academy of Sciences, H-1083 Budapest, Szigony utca 43, Hungary

**Keywords:** SYSNEURO, MOLNEURO, SIGNALING

## Abstract

Electrical synapses between interneurons contribute to synchronized firing and network oscillations in the brain. However, little is known about how such networks respond to excitatory synaptic input. To investigate this, we studied electrically coupled Golgi cells (GoC) in the cerebellar input layer. We show with immunohistochemistry, electron microscopy, and electrophysiology that Connexin-36 is necessary for functional gap junctions (GJs) between GoC dendrites. In the absence of coincident synaptic input, GoCs synchronize their firing. In contrast, sparse, coincident mossy fiber input triggered a mixture of excitation and inhibition of GoC firing and spike desynchronization. Inhibition is caused by propagation of the spike afterhyperpolarization through GJs. This triggers network desynchronization because heterogeneous coupling to surrounding cells causes spike-phase dispersion. Detailed network models predict that desynchronization is robust, local, and dependent on synaptic input properties. Our results show that GJ coupling can be inhibitory and either promote network synchronization or trigger rapid network desynchronization depending on the synaptic input.

## Highlights

► Gap junctions (GJ) are made between Golgi cell dendrites and depend on Cx36 ► GJs mediate surround inhibition by preferentially propagating spike AHPs ► Sparse synaptic excitation desynchronizes GJ-coupled Golgi cell networks ► Desynchronization arises from network heterogeneity and is stimulus dependent

## Introduction

Brain oscillations reflect the synchronized activity of groups of neurons and can contribute to neural computation in several ways (Buzsaki and Draguhn, 2004; Sejnowski and Paulsen, 2006). Sensory information can be encoded as spike times within the oscillation cycle (phase code, O'Keefe and Recce, 1993) and oscillations in the olfactory system contribute to sparse odor representations and odor discrimination (Stopfer et al., 1997). Moreover, spatially distributed information processing is thought to be enabled by long-range oscillations, which temporally “bind” segregated neuronal ensembles (Singer and Gray, 1995). These important potential roles for network oscillations have triggered a number of studies investigating the underlying mechanisms. These show that inhibitory interneurons play a central role in synchronizing firing within networks and that electrical synapses between interneurons enhance synchrony. This has been particularly well studied in the neocortex, where inhibitory interneurons of particular subtypes are electrically coupled forming discrete network modules (Galarreta and Hestrin, 1999; Gibson et al., 1999; Szabadics et al., 2001; Tamas et al., 2000).

Electrical coupling promotes synchronization of membrane potential across cells because gap junctions (GJs) pass a current that is proportional to potential difference, thereby tending to equalize the voltage between coupled cells. Electrical coupling between neurons have been observed in many regions of the mammalian brain including the inferior olive (Devor and Yarom, 2002; Llinas et al., 1974; Van Der Giessen et al., 2008), thalamus (Hughes et al., 2002; Landisman et al., 2002), hippocampus (Kosaka and Hama, 1985), neocortex (Galarreta and Hestrin, 1999; Gibson et al., 1999), olfactory bulb (Schoppa and Westbrook, 2002), and cerebellar cortex (Dugue et al., 2009; Mann-Metzer and Yarom, 1999; Sotelo and Llinas, 1972). GJs have been shown to enhance network oscillations (Beierlein et al., 2000; Deans et al., 2001; Hormuzdi et al., 2001; Hughes et al., 2004; Traub et al., 2001; Whittington et al., 1995). But in most of these studies synchronized activity occurred in the absence of excitatory drive, in cells driven by steady input current or where exogenous transmitters were used to generate a tonic asynchronous excitatory network drive (Beierlein et al., 2000; Buhl et al., 1998; Devor and Yarom, 2002; Dugue et al., 2009; Galarreta and Hestrin, 1999; Gibson et al., 1999; Hormuzdi et al., 2001; Hughes et al., 2004; Long et al., 2004; Mann-Metzer and Yarom, 1999). In contrast, relatively little is known about how electrically coupled networks respond to synchronous synaptic excitation.

Network oscillations, such as the alpha and μ EEG rhythms (8–12 Hz) in humans and the 6-10 Hz somatosensory (theta) rhythm in rodent cortex and cerebellum, rapidly desynchronize in response to cognitive activity and external sensory stimuli (Buzsaki, 2006; Courtemanche and Lamarre, 2005; O'Connor et al., 2002; Pfurtscheller and Lopes da Silva, 1999; Poulet and Petersen, 2008; Wiest and Nicolelis, 2003). This is thought to depend on thalamo-cortico-cerebellar circuits but the cellular mechanisms are poorly understood (Buzsaki, 2006; Pfurtscheller and Lopes da Silva, 1999). It has been suggested that for random stimuli, suppression of such low-frequency oscillations increases the sensitivity of the network to sensory stimuli and that this can be modulated by attention (Schroeder and Lakatos, 2009). It has also been proposed that desynchronization of gamma oscillations reflects a process of active uncoupling of neural ensembles to allow the emergence of new ensembles, which may be necessary to proceed from one cognitive state to another (Rodriguez et al., 1999). In addition, abnormal synchronization and desynchronization of network oscillations of different frequencies have been associated with several neurological disorders, including epilepsy, schizophrenia, dementia and Parkinson's disease (Schnitzler and Gross, 2005). In contrast to the extensive body of work on network synchronization little is known about how networks desynchronize.

The Golgi cell (GoC) network in the input layer of the cerebellar cortex is a particularly attractive model system for studying the behavior of electrically coupled networks, because GoCs do not form inhibitory chemical synapses with one another, allowing electrical transmission to be studied in isolation (Dugue et al., 2009). GJs have been shown to promote GoC synchronization, causing a rhythmic inhibition of the granule cell population (Dugue et al., 2009). Moreover, GoCs are sparsely innervated by excitatory mossy fiber (MF) synapses, which can be selectively activated in acute slices (Kanichay and Silver, 2008). GoCs have also been studied extensively in vivo: the observation that GoCs fire in phase with local field potential oscillations at theta frequency (∼8 Hz) in freely roaming rodents during quiet attentiveness (Dugue et al., 2009) is consistent with synchronization via GJ coupling. However, the rapid, behavior-dependent disappearance of these oscillations (Courtemanche et al., 2002; Hartmann and Bower, 1998; O'Connor et al., 2002; Pellerin and Lamarre, 1997), together with the highly unpredictable nature of GoC responses during sensory evoked input (Holtzman et al., 2006a, 2006b; Prsa et al., 2009; Simpson et al., 2005) is less easy to explain. For example, following tactile stimulation of the face (Vos et al., 1999), whiskers (Holtzman et al., 2006b; Vos et al., 1999), and limbs (Holtzman et al., 2006a, 2006b) or during eye saccade movements (Prsa et al., 2009), spontaneously active GoCs can either pause their firing, or less commonly, fire a burst of action potentials followed by a pause. The inhibitory pause-only behavior is puzzling because their input is predominantly excitatory, through direct feed-forward input from MFs (Eccles et al., 1967; Kanichay and Silver, 2008; Vos et al., 1999), feedback excitation from granule cell axons (Dieudonne, 1998; Vos et al., 1999), and possibly through climbing fiber input (Eccles et al., 1967).

Here we show that Connexin-36 is necessary for electrical coupling between GoCs and that GJs are mainly located on the apical dendrites. While synchronization of the GoC network at theta frequency is the “default state” in the absence of synaptic input and during asynchronous synaptic input (Dugue et al., 2009), our experiments and modeling show that sparse synaptic excitation triggers local and transient network desynchronization through GJ mediated surround inhibition. Moreover, the firing behavior of GoCs recorded in acute slices during synchronous excitatory synaptic input and the behavior predicted from anatomically and electrophysiologically detailed network models can account for much of the variability in GoC behavior observed in vivo.

## Results

### Excitatory Synaptic Input Can Excite or Inhibit Golgi Cells

**Figure 1:**
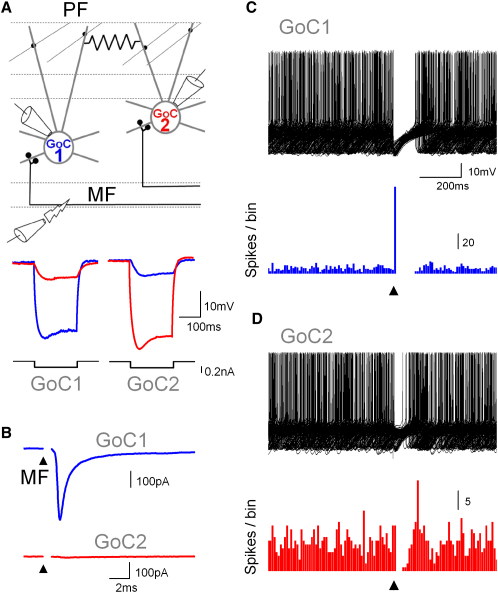
Mossy Fiber Stimulation Can Excite or Inhibit Golgi Cell Firing (A) Schematic diagram of paired Golgi cell (GoC) recording configuration with mossy fiber (MF) stimulation (PF; parallel fiber input). Voltage responses recorded in a cell pair in response to injected current pulses (−0.2 nA) in one of the cells. The coupling coefficient (CC) was 19.4%. (B) MF stimulation produced an EPSC only in GoC1 (V_hold_ = −70 mV) in the presence of 10 μM gabazine and 0.5 μM strychnine to block inhibition. (C) Superimposed voltage recordings showing firing of GoC1 during single shock MF stimulation (same stimuli as in B), which reliably evoked a spike followed by a pause of firing. Bottom panel shows spike histograms (10 ms bins). (D) Superimposed voltage recordings showing firing of GoC2 during the same trials as (C), together with spike histograms below. GoC2 responded to MF input into GoC1 with an inhibitory pause. See also Figure S1.

To investigate the functional properties of the Golgi cell (GoC) network we performed whole-cell recordings from pairs of GoCs in acute slices of the cerebellar cortex from 2- to 4-week-old mice (Figure 1A; mean somata distance: 69 ± 2 μm, n = 174 pairs). We tested for electrical coupling by hyperpolarizing them to prevent spontaneous firing and examining their voltage responses to current pulses injected into only one of the cells (Figure 1A). The coupling coefficient (CC; the ratio of voltage changes in the cell pair) was 8.2% ± 0.4% across all pairs (n = 174). We found no evidence for chemical inhibitory synapses between GoC pairs in our 174 paired recordings in agreement with a previous report (Dugue et al., 2009). To examine how coupled cells respond to excitatory synaptic input, MFs were activated with a patch electrode placed in the white matter (Kanichay and Silver, 2008) (Figure 1A). The electrode position and strength of the stimulation pulse were adjusted to reliably activate a large, short-latency EPSC (250 ± 40 pA, n = 9) in only one of the GoCs (Figure 1B, somata distance: 52 ± 5 μm; CC: 17.3% ± 2.1%, n = 9). EPSCs of this amplitude, corresponding to ∼4 MF inputs (Kanichay and Silver, 2008), could routinely be evoked in only one of the cells, suggesting that MF innervation of GoCs is sparse. To rule out the involvement of chemical inhibitory synaptic inputs, the GABA_A_R antagonist gabazine (10 μM) and the glycine receptor antagonist strychnine (0.5 μM) were added to the ACSF. After switching to current-clamp, the same stimulation protocol evoked a precisely timed spike followed by a pause in the cell receiving the direct MF input (Figure 1C, mean pause duration: 245 ± 18 ms, n = 9). In contrast, MF input evoked only an inhibitory pause in the spontaneous firing of the coupled cell (Figure 1D, mean pause duration: 112 ± 13 ms, tonic firing frequency of GoC2: 5.7 ± 0.4 Hz, n = 9). The EPSCs together with these excitatory and inhibitory responses in GoCs were blocked by the glutamate receptor antagonists NBQX (10 μM) and AP-5 (50 μM, n = 9), indicating that they were triggered by excitatory synaptic input and not direct stimulation of the GoC axon.

GoCs also receive excitatory input from granule cells via the parallel fibers (PFs). We examined whether PFs also produced mixed GoC responses. Since the dendrites of the GoC pairs only overlap partially, it was possible to activate distinct PF inputs by stimulating in the molecular layer (Figure S1A, somata distance: 64 ± 9 μm, CC = 12% ± 2%, n = 13). We evoked large PF EPSCs in only one of the coupled GoCs (Figure S1B, mean EPSC 235 ± 25 pA, n = 13). In current clamp, PF stimulation caused a spike followed by a pause in the synaptically excited GoC (Figure S1C, mean pause duration 214 ± 16 ms, n = 13) and a pause in the firing of the other cell (Figure S1D, mean duration: 67 ± 11 ms, tonic firing frequency: 6.0 ± 0.3 Hz, n = 13). These results indicate that both sparse MF input and beams of PF input can excite or inhibit electrically coupled GoCs.

### Potential Contribution of Chemical Inhibition to the Pause in Golgi Cell Firing

Previous studies have suggested that GoC pauses may arise from chemical synaptic inhibition from Lugaro, Purkinje, stellate, or basket cells (Holtzman et al., 2006b). To test whether MF-evoked chemical inhibition could also contribute to GoC pauses, we carried out experiments in the absence of blockers for chemical inhibition. GoCs were voltage clamped at the reversal potential for excitatory synaptic input. Strong stimulation of the white matter (10 pulses at 100 Hz) evoked NBQX-sensitive disynaptic IPSCs in only 15% of the GoCs tested in horizontal (3 out of 21 cells) or sagittal slices (one out of five cells). When present, inhibitory conductances were relatively weak (0.9 ± 0.29 nS, n = 4). In contrast, Purkinje cells often showed larger disynaptic inhibition under the same conditions (62% of cells, 7.8 ± 3.9 nS, n = 8, horizontal slices). In current clamp, we were unable to selectively activate IPSPs in GoCs without evoking large EPSPs that trigger spikes. We also examined whether GABA_B_ receptor activation contributed to GoC inhibition. However, the selective agonist baclofen (50-100 μM) only produced a small outward current (10-20 pA) that was blocked by the antagonist CGP55845 (10-20 μM) in 3 of the 18 GoCs tested, indicating that only a small subset of GoCs express these receptors postsynaptically. Finally, we used the GJ blocker mefloquine (Cruikshank et al., 2004) to directly test the hypothesis that GJs mediate MF-evoked inhibitory effects in GoCs. Mefloquine reduced GoC coupling with its effect taking ∼1 hour (Figures S2A and S2B) and abolished inhibitory potentials evoked by bursts of MF input (Figures S2C and S2D, n = 5), thereby demonstrating that they are dependent on intact electrical coupling. These data suggest that the MF-evoked inhibitory pauses in GoCs are mediated by electrical synapses rather than disynaptic chemical inhibition.

### Time Course of Electrical Excitation and Inhibition in Golgi Cells

**Figure 2:**
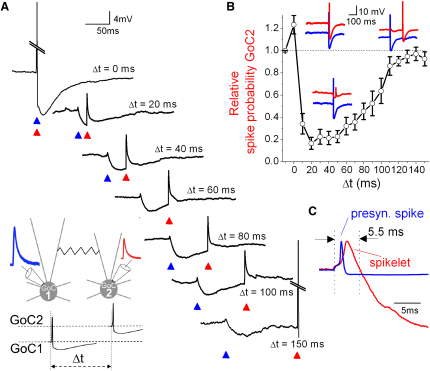
Time Course of Electrical Excitation and Inhibition in Golgi Cells (A) (Inset) Schematic diagram showing recording from two Golgi cells (GoCs) and the timing protocol of injected EPSC-like current waveforms (iEPSC). The iEPSC in GoC1 was scaled to reliably evoke a spike, while the iEPSC in GoC2 was scaled to evoke spikes with a probability of ∼0.7. The coupling coefficient (CC) was 16.3%. A small steady negative current (10–20 pA) was injected to prevent spontaneous firing. From top to bottom: individual voltage responses of GoC2 for different intervals (Δt) between iEPSC injection into the two cells. At Δt = 0 and 150 ms the iEPSC caused a spike (truncated for clarity), but at intermediate times the GJ potential inhibited spike generation in GoC2. (B) Relative spike probability of GoC2 as a function of Δt (for Δt > 100 ms, n = 4 cell pairs; for Δt < 100, n = 9 cell pairs). Insets show examples of the membrane voltage traces of GoC1 (blue) and GoC2 (red) for Δt of 0, 40, and 130 ms. Note that GoC2 fails to spike when Δt = 40 ms. Data represent mean ± SE. (C) Example of a spike in GoC1 and the spikelet in GoC2, normalized to the peak. See also Figure S2.

To examine the time course of electrical transmission we replaced MF stimulation with direct current injection, since this eliminated potential synaptically evoked network effects. To generate EPSP-like depolarizations in GoCs, we injected a previously characterized MF EPSC current waveform (iEPSC) into the soma through the patch pipette (Kanichay and Silver, 2008). A suprathreshold iEPSC in one of the GoCs evoked a pause in the coupled GoC (104 ± 7 ms, n = 14) of similar duration to those evoked by MF stimulation (p > 0.05, Wilcoxon signed rank test), indicating that inhibition is comparable with these two approaches. To examine the time course of inhibition, iEPSCs, injected into both GoCs, were scaled in amplitude to generate a spike probability of 1 across trials in cell 1 (Figure 2A, for reliable spike generation) and a spike probability of 0.69 ± 0.06 (n = 9) in cell 2 (Figure 2A; to allow bidirectional modulation of the spike probability). When currents were injected in both cells simultaneously the spike probability in cell 2 was increased by 23% ± 8% (Figure 2B; CC: 13.2% ± 1.8%, n = 9) due to the initial, small depolarizing component of the GJ potential (“spikelet”; Galarreta and Hestrin, 2001a; Figure 2C). In contrast, delaying the timing of the current injected into cell 2 by only 10 ms produced a profound inhibition of the spike probability (Figures 2A and 2B). Spike probability was maximally inhibited (83% ± 5%, n = 9) at an interval of 20 ms and had relaxed back to the control level when the interval was increased to 120 ms (Figure 2B). The close correspondence between the direction and time course of spike probability and the time course of the spikelet and hyperpolarizing components of the GJ potential suggests that synaptically evoked pauses in firing are caused by the propagation of the spike afterhyperpolarization (AHP) through GJs. These results show that electrical synapses in GoCs are predominantly inhibitory.

### Spike Desynchronization Triggered by Sparse Excitatory Synaptic Input

**Figure 3:**
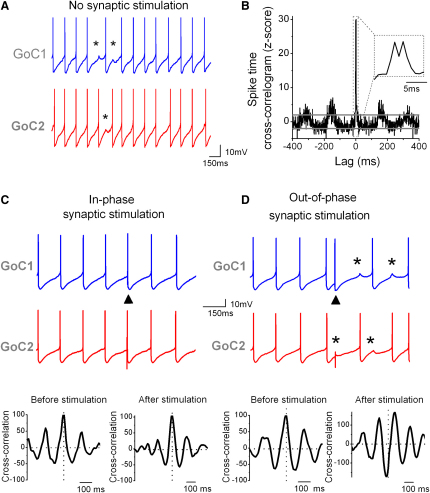
Sparse Synchronous Synaptic Input Can Cause Spike Desynchronization (A) Voltage traces from a Golgi cell (GoC) pair (coupling coefficient [CC]: 23.1%) in the absence of synaptic stimulation. Spikes were synchronized, but they occasionally skipped a cycle (asterisks) producing transient antiphase firing. (B) Cross-correlogram of spike times for the same cells as in (A). Horizontal lines indicate confidence intervals. Inset shows the central peak at an expanded timescale. (C) Voltage traces from another GoC pair (CC: 19.4%). Stimulation of mossy fiber (MF) input into GoC1 (arrowhead) in phase with a spike did not perturb spike synchronization between GoC1 and GoC2. (Bottom panels) Cross-correlation of the spike time distribution of both cells across multiple repeated trials, in a 300 ms time window before stimulation (left), and in a 400 ms time window after the synaptic stimulation (right). Only sweeps where both GoCs showed spike synchrony before stimulation were included. Inhibitory synaptic transmission was blocked with 10 μM gabazine and 0.5 μM strychnine. (D) Out-of-phase stimulation of the same MF input into GoC1 caused antiphase firing and short-term spike desynchronization, as indicated by the trough in the cross-correlation plot below. Note that after stimulation both cells mutually inhibit each other by the propagation of their spike AHP (asterisks) as can be seen occurring also spontaneously in (A).

Electrical synapses are thought to be a major determinant of network synchronization in the CNS. Consistent with this view, coupled GoCs (CC > 4.7%) showed significant spike synchronization in the absence of synaptic stimulation (Figures 3A and 3B, n = 30) as reported previously for low levels of asynchronous synaptic input (Dugue et al., 2009). The narrow double peak in the spike-time cross-correlogram indicates spike synchronization with millisecond precision (Figure 3B, inset). Although precise, this coupling appears loose, because even in the quiescent state spikes occasionally skip cycles (Figure 3A, asterisks), often producing alternation of spikes between GoC pairs (Dugue et al., 2009). To investigate whether such transient antiphase behavior or other forms of desynchronization can be induced by sparse excitatory synaptic inputs, we stimulated an input into one of the cells at different phases in the spiking cycle. Precisely timed synaptic input that occurred in-phase with the synchronized spikes preserved spike synchrony between the two GoCs (Figure 3C), as shown by the clear central peak in the cross-correlation of the spike time distribution of both cells across multiple repeated trials (Figure 3C, bottom). In contrast, synaptic input that occurred out-of-phase with spikes desynchronized the GoC pair and drove the two cells into an antiphase pattern of spiking (Figure 3D, CC: 24.1 ± 1.8, n = 5) and produced a central trough in the cross-correlation rather than a peak. These experiments demonstrate that a temporally precise, sparse excitatory synaptic input can trigger antiphase firing in pairs of electrically coupled cells. This suggests that sparse MF input could cause desynchronization of spike times in the GoC network.

### Golgi Cell Responses to Bursts of Synaptic Input

**Figure 4:**
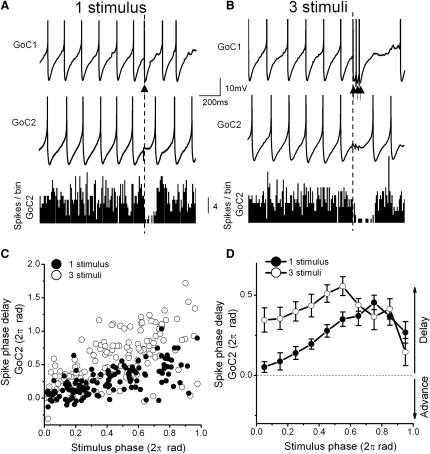
Golgi Cell Inhibitory Responses to Bursts of Synaptic Input (A) Voltage traces from a Golgi cell (GoC) pair (coupling coefficient [CC]: 23.9%) during mossy fiber (MF) input into one of the cells. Inhibitory synaptic transmission was blocked by 10 μM gabazine and 0.5 μM strychnine. A single MF stimulus reliably evoked a spike in GoC1, while causing a pause in the firing of GoC2. (Bottom panel) Spike histogram of GoC2 (10 ms bins). (B) In the same cell pair, three stimuli at 33 Hz evoked multiple spikes in GoC1 causing longer pauses in firing of GoC2. (C) Individual phase response curves for cell pair in (A) and (B), where the average interspike interval corresponds to a full cycle (2π radians). MF stimulation delayed the phase of spikes in GoC2 as a function of the stimulation phase for one and three MF stimuli. (D) Average phase response curves for one and three MF stimuli (n = 5 cell pairs; p < 0.05 when stimulus phase < 0.65). Data represent mean ± SE. See also Figure S3.

Since some types of sensory stimuli can be encoded in high frequency bursts of MF firing (Rancz et al., 2007), we examined how GoCs respond to four MF stimuli at 100 Hz. This brief burst typically evoked a single AP in the directly innervated GoC and triggered an inhibitory pause in the coupled GoC with a duration comparable to that for a single MF stimulus (135 ± 7 ms, n = 6; p > 0.05, data not shown). In vivo recordings also show that brief tactile stimuli to the skin (Holtzman et al., 2006a, 2006b; Jorntell and Ekerot, 2006), whiskers (Vos et al., 1999), or eye saccades (Prsa et al., 2009) can be encoded in GoCs as brief bursts of APs followed by pauses in their firing. To investigate how the GoC network responds to such input, we compared the effect of a single MF stimulus that triggered one AP, with three MF stimuli at 33 Hz which evoked a burst of two to three spikes in the cell receiving the input (Figures 4A and 4B) (Prsa et al., 2009). The bursts of GoC spikes resulted in a significantly longer postsynaptic depression (178 ± 18 ms, range 140-221 ms) than that mediated by a single AP (116 ± 7 ms, range 90-130 ms; n = 5, p = 0.002). These longer pauses were not caused by differences in tonic firing frequency of the postsynaptic cells in the two groups (5.7 ± 0.5 Hz and 5.9 ± 1 Hz, respectively, p = 0.46, n = 5). Likewise, repetitive stimulation of PF input (three stimuli at 20 Hz) also caused significant longer pauses than a single PF stimulus (one stimulus: 67 ± 11 ms, n = 13; three stimuli: 210 ± 9 ms, n = 4, p = 0.0005, data not shown). This suggests that the temporal pattern of synaptic stimuli could be encoded in the duration of pauses in firing within the electrically coupled GoC network.

To investigate whether the phase at which the synaptic input occurred in the spike cycle influenced the duration of the pause induced in the electrically coupled GoCs, we constructed phase response curves of both the single-stimulus and three-stimuli experiments (Figure 4C). On average, the spike in the cell not receiving synaptic input occurred later in the cycle (phase delay; i.e., longer pause) as the timing of the synaptic input was delayed, reaching a peak at ∼0.7 of the full cycle (Figure 4D). However, this spike-phase delay reduced sharply as the timing of the synaptic input neared the end of the spike cycle. The three-stimuli synaptic input produced larger spike delays earlier on in the spike cycle, but after 0.65 of the full spike cycle the one and three shock protocols became indistinguishable (Figure 4D). As expected, the pause duration and spike-phase delay also depended on the spontaneous firing frequency of the GoCs and the electrical coupling strength between them (Figures S3A–S3D). Nevertheless, this phase analysis shows that timing of excitatory synaptic input and the temporal pattern of presynaptic stimuli influence the duration of the pause in spiking induced in neighboring electrically coupled GoCs.

### Molecular Identity and Location of Gap Junctions between Golgi Cells

**Figure 5:**
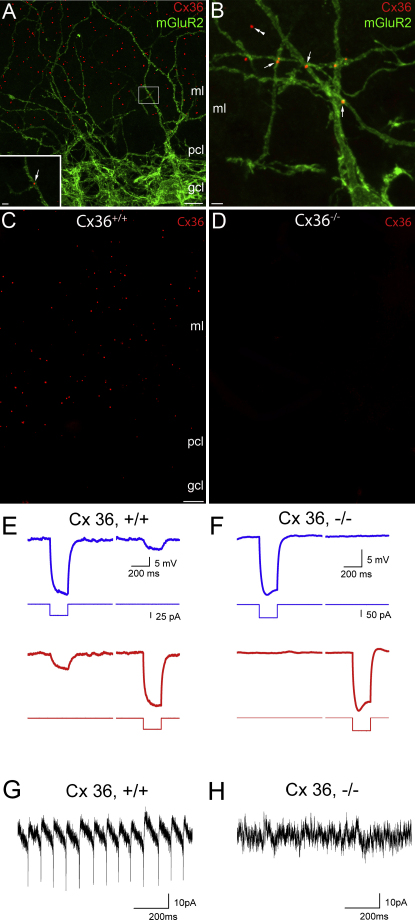
Connexin-36 Mediates Electrical Coupling between Golgi Cell Dendrites (A and B) Double immunofluorescent reactions for Cx36 (red) and the selective Golgi Cell (GoC) marker mGluR2 (green) in mouse cerebellar cortex. Some Cx36-immunoreactive puncta were detected at the intersections of two mGluR2-immunopositive dendrites (arrows). The majority of Cx36-immunopositive puncta were not associated with mGluR2-immunopositive dendrites (double arrowheads). Inset in (A) is a high-magnification view of the boxed area. Inset in (A) is a single confocal section. Rest: maximum intensity Z projection of (A, 11; B, 8) confocal sections (at A, 2 μm; B, 0.5 μm separation). (C and D) Immunofluorescent labeling of Cx36 in the cerebellar cortex of a wild-type mouse (*Cx36*^+/+^, C) and a Cx36 knockout littermate (*Cx36*^−/−^, D). (E and F) Voltage responses of a GoC pair from a *Cx36*^+/+^ (E) and a *Cx36*^−/−^ mouse (F) in response to injected current pulses in one of the cells. A small hyperpolarizing current (5–60 pA) was applied to stop the cells from spontaneous firing. (G and H) Voltage-clamp recordings with a Cs-gluconate-based internal solution containing QX314 and tetraethylammonium (TEA) showing rapid inward spikelets followed by slower outward currents from single GoCs. Cells were held depolarized at −30 to +30 mV. Spikelets were observed in *Cx36*^+/+^ but not in *Cx36*^−/−^ mice. Scale bars: (A, C, and D) 10 μm, (A, inset, and B) 2 μm, (C) and (D) are at the same magnification. pcl, Purkinje cell layer; gcl, granule cell layer; ml, molecular layer. See also Figure S4.

Little is known about the molecular properties and location of electrical synapses in GoCs. To investigate this, we used immunofluorescent labeling to identify GoCs and to localize GJ proteins. We used mGluR2 as a molecular marker of GoCs because, in the cerebellar cortex, it is exclusively expressed by GoCs (Ohishi et al., 1994). Figure 5 shows immunofluorescent reactions for mGluR2 labeling in green and Connexin 36 (Cx36) in red. Punctuate Cx36 labeling was observed mainly in the molecular layer of both mice (Figures 5A–5C) and rats (Figures S4A and S4B). In contrast to wild-type animals, no detectable Cx36 labeling was found in slices from Cx36 knockout mice (Figure 5D), demonstrating the specificity of our immunoreactions. Immunoreactive puncta for Cx36 were often found at the intersection of mGluR2-positive dendrites originating from different GoCs (Figure 5B). The majority of these mGluR2-associated Cx36 puncta were observed on apical dendrites in the molecular layer and fewer on basolateral dendrites in the granule cell layer. This may reflect a specific targeting of Cx36 or that most of the GoC dendrites are situated in the molecular layer (Dieudonne, 1998; Kanichay and Silver, 2008). It is important to note that most of the Cx36 immunopositive puncta in the molecular layer were not associated with mGluR2-positive dendrites. Because mGluR2 only labels approximately 80% of GoCs (Simat et al., 2007), some of those puncta might be between mGluR2 negative GoCs or between stellate or basket cells (Mann-Metzer and Yarom, 1999; Sotelo and Llinas, 1972).

To test whether Cx36 forms functional GJs between GoC dendrites, we carried out electrophysiological experiments on Cx36 knockout mice (Deans et al., 2001). Typical voltage responses to a current pulse injected into one GoC are shown for a wild-type and a knockout animal in Figures 5E and 5F. In contrast to the high connection probability of wild-type littermates (83%; n = 6), we found no instances of electrical coupling in the knockout animals in seven paired recordings. This result was confirmed in a larger sample of single GoC voltage-clamp recordings, where spikelets were absent in KO mice (WT: 75% [n = 16]; KO: 0% [n = 17]; Figures 5G and 5H). These results demonstrate that Cx36 is required to form functional GJs between GoCs, but do not rule out that other connexin subunits, such as Cx45 (Van Der Giessen et al., 2006) are also present in GoCs.

**Figure 6:**
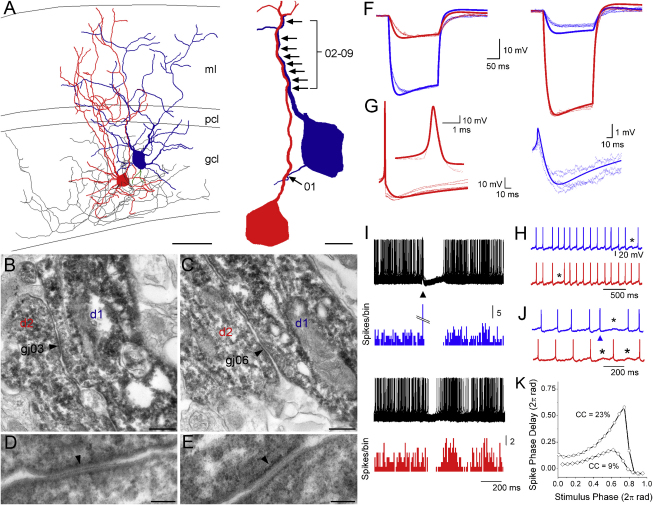
Electron Microscopic Localization of Gap Junctions between Two Golgi Cells and Compartmental Modeling of Their Electrophysiological Behavior (A) (Left) Light microscopic reconstruction of two electrically coupled Golgi cells (GoCs) previously whole-cell recorded and filled with biocytin (GoC1: soma and dendrites, blue; axon, green; GoC2: soma and dendrites, red; axon, black). (Right) High-magnification view of the gap junction locations (GJs, arrows). Other dendrites are not shown for clarity. (B and C) Electron micrographs of two GJs (gj03 and gj06) formed by dendrites d1 and d2. (D and E) High-magnification images of the GJs (arrowheads) shown in (B) and (C). (F–K) Simulations with the reconstructed morphologies of the coupled GoC pair. The nine GJs were inserted at positions determined by electron microscopy. (F) Comparison of the model and the experimental responses (from the same GoC pair) to 200 ms current pulses (−200 pA). Throughout (F) and (G), thin lines are five experimental responses; thick lines are the model predictions. During the experiments and in the model, steady negative current was applied to stop the cells from rhythmic firing (typically −5 to −100 pA, baseline V_m_ in model and experiment: ∼−55 mV). The conductances of the GJs were adjusted to match the voltage attenuation from the blue to the red GoC (left panels; each GJ = 130 pS). Note that this did not match perfectly the attenuation from the red to the blue GoC (right panels). Experimental CC from blue to red = 28.2%, CC from red to blue = 15.4%. (G) (Left) Experimental and model spikes and AHPs (during spontaneous firing at ∼8 Hz). Inset shows spikes on an expanded timescale. (Right) Experimental and model GJ potentials. (H) Model voltage responses showing spike synchrony in the absence of synaptic input and occasional missed spikes (asterisks). (I) Model spike train responses (black, 150 superimposed traces) and spike histograms (10 ms bins) of both GoCs. GoCs were spontaneously spiking at ∼8 Hz. Simultaneous activation of 20 mossy fiber (MF) synapses on the blue GoC (arrowhead) reliably evoked a spike followed by a pause, while only causing a pause in the firing of the red GoC. (J) Simulations showing that out-of-phase MF input (arrow) to the blue GoC caused transient antiphase firing in the cell pair. (K) Model phase response curves of red GoC when the blue GoC is stimulated with MF input (as in I and J). In this simulation the conductance of the GJs was adjusted to give a CC of 9% and 23% in order to compare with experimental data in Figure S3C; note that due to the smaller bin-width of the model data, the phase advance for spikes later in the cycle is more pronounced than for the experimental data. ml, molecular layer; pcl, Purkinje cell layer; gcl, granule cell layer. Scale bars: (A) 50 μm, (A, inset) 10 μm, (B and C) 250 nm, (D and E) 50 nm. See also Table S1 and Figure S5.

To examine the location and number of GJs on GoCs, we reconstructed two biocytin labeled GoC pairs and performed electron microscopic analysis of their connections. Light microscopy was used to determine where the cells made close dendritic appositions (Figure 6A). Electron microscopy (EM) analysis of these regions, revealed multiple putative GJs. GJs were identified on the basis of tight appositions of the cell membranes and a characteristic fine electron dense line between them (Figures 6B–6E). Of the nine GJs identified in this cell pair, eight were located between the apical dendrites and one between a basolateral and an apical dendrite (Figure 6A, right). This pattern was also seen in the second reconstruction, which had two GJs, one located between apical dendrites and the other between basolateral dendrites. The presence of GJs between GoC dendrites was also confirmed in preparations that lack electron dense diaminobenzidine precipitates in the dendrites. This involved pre-embedding immunogold localization of mGluR2 and identifying GJs between mGluR2-positive GoC dendrites at the EM level (Figure S5). These results demonstrate that electrical coupling between GoCs is mediated by multiple GJs located on their dendrites.

### Properties of Electrical Synapses Reproduced with an Anatomically and Biophysically Constrained Model of a Golgi Cell Pair

To test whether the diverse GoC behavior we observed experimentally could solely arise from an interaction between excitatory synaptic input, spontaneous GoC firing, and electrical coupling, we built a model of one of our cell pairs. The Neurolucida files of the two cells shown in Figure 6A were imported into neuroConstruct (Gleeson et al., 2007), and converted into an electrical compartment representation including full dendrites and all recovered axon (4822 and 2019 segments for cell 1 and 2, respectively). Thirteen Hodgkin-Huxley and Markov-type active conductances and Ca^2+^ buffer mechanisms from a recently developed model of a GoC (Solinas et al., 2007) that meticulously reproduced previous experimental data from rat cerebellum (Forti et al., 2006) were implemented in our multicompartment models, because its behavior was very similar to our data from mouse (Table S1). We did not change the active conductances or their densities in the model. We only altered the leak conductance of each cell to match the experimentally measured input resistance (Figure 6F). Electrical coupling was implemented by placing nine GJ conductances at the exact positions observed with EM. The size of the GJ conductances was adjusted so that the steady-state voltage responses to negative current steps matched as closely as possible those found experimentally (Figure 6F). The model reproduced well the experimental data including spike shape, AHP amplitude and duration and GJ potential (Figure 6G).

We then investigated whether our model could reproduce the experimentally observed firing behavior of GoCs in the presence and absence of excitatory synaptic input. To do this, MF-like synapses were randomly distributed along the basolateral dendrites and the amplitudes and time courses of the conductance waveforms were set to experimentally determined values (Kanichay and Silver, 2008). Moreover, noise was added to reproduce in vitro interspike interval (ISI) variability (Solinas et al., 2007). In the absence of synaptic input the spike times in the model cells became synchronized and occasional spontaneous pauses were observed as for the real cells (Figure 6H). Synchronous activation of MF input into one GoC triggered a spike in that cell (e.g., blue cell in Figure 6I), but only produced a pause in firing in the coupled GoC (red cell in Figure 6I). Furthermore, out of phase MF input into the blue GoC triggered antiphase firing in the model cell pair (Figure 6J). Moreover, when the CC was adjusted to match the average values of the upper and lower half of the CC population data (Figure S3C) the cell pair exhibited phase response curves with similar properties to real GoCs (compare Figures 6K and S3C). These simulations show that our anatomically and biophysically detailed model of a GoC pair can reproduce both the spontaneous behavior of GoC pairs and the excitatory and inhibitory firing behavior of GoCs during sparse excitatory synaptic input. This suggests that excitatory synaptic input together with the morphology, intrinsic conductances and the electrical coupling between GoC pairs is sufficient to generate the behavior we observed experimentally.

### An Anatomically and Biophysically Detailed Model of the Electrically Coupled Golgi Cell Network

**Figure 7:**
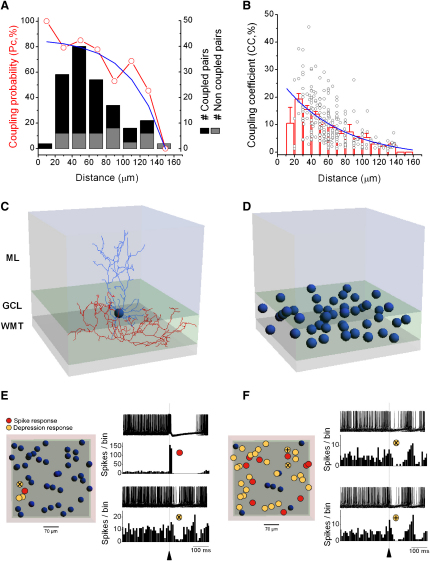
Sparse Excitatory Inputs Evoke Gap Junction-Mediated Surround Inhibition in a GoC Network Model (A) Experimentally measured coupling probabilities (P_c_) between pairs of Golgi cells (GoCs) as a function of distance between their somata (136 pairs). The P_c_ is fitted with a Boltzmann function (blue, y = −1745 + 1836/{1 + exp[(distance − 267)/39]}). Histogram shows the number of coupled and noncoupled GoC pairs (20 μm bins). (B) Experimentally measured coupling coefficient (CC, circles) as a function of distance between somata (174 pairs). Histogram shows the binned CC (10 μm bins). CC data are fitted with a single exponential decay function [blue, y = −2.3 + 29.7 ^∗^ exp(–distance/70.4)]. Column bars represent mean ± SE. (C) The 3D volume of the network model comprising white matter tract (WMT) granule cell layer (GCL) and molecular layer (ML) together with reconstructed GoC used in the network model (soma and dendrites, blue; axon, red). (D) Forty-five randomly distributed GoCs in a modeled slab of GCL of 350 × 350 × 80 μm; dendrites and axons are omitted for clarity. (E) (Left) Top view of the 3D model. Temporally precise synaptic input to a random GoC (red, Experimental Procedures) evoked an excitation followed by a pause while three neighboring GoCs (yellow) that did not receive synaptic input showed pause-only responses in their spontaneous spiking. (Right) Top two panels (red cell): superimposed spike trains (100 traces) and spike histogram (10 ms bins). Bottom two panels show a typical yellow cell pause. (F) (Left) The same synaptic input as in (E) to ten random GoCs (∼22% of the network) triggered an excitation followed by a pause in the red GoCs while 27 GoCs responded only with a pause in spontaneous spiking (yellow). (Right) Top two panels: the majority of inhibited GoCs responded with a pure pause (yellow, “x”); bottom two panels: a minority of inhibited GoCs showed a small peak before the pause in the spike histogram (yellow, “+”). See also Figure S6.

To test whether synaptic input triggers network desynchronization and to explore the spatial properties of network dynamics, we extended our two-cell model to a larger 3D GoC network model. Construction of a GoC network model required information about the spatial properties of electrical coupling. We therefore calculated the coupling probability and CC as a function of radial distance between the somata from our data set. The coupling probability decreased sharply at distances of > 100 μm and was close to zero at 150 μm (Figure 7A; Dugue et al., 2009). The CC decreased exponentially with a space constant of 70 μm (Figure 7B). We used neuroConstruct (Gleeson et al., 2007) to randomly place GoC somata within a 350×350×80 μm volume (Figures 7C and 7D) at a measured density of 4607 ± 166 cells/mm^3^ (n = 4 rats; A.L., Z.N., R.A.S., unpublished data). Algorithms within neuroConstruct determined probabilistically whether any two cells were electrically coupled according to the fit of the spatial dependence of the coupling probability (Figure 7A) and, if coupled, the connection was made randomly on the dendritic tree. The conductance of the electrical coupling between the two cells was then determined from the spatial dependence of the CC (Figure 7B) and the relationship between CC and coupling conductance (Figure S6A). Since GoCs have a far larger apical than basal dendritic tree, most electrical connections were made in the molecular layer in agreement with the anatomical data (Figures 5A and 5B). For each GoC in the network, MF excitatory synaptic inputs were randomly distributed over the basolateral dendrites in the granule cell layer (GCL) and PF inputs were distributed over ascending dendrites within the molecular layer. No chemical inhibitory synaptic inputs were included in the model. Background synaptic noise similar to that observed in vivo (Chadderton et al., 2004; Rancz et al., 2007) and heterogeneity in intrinsic GoC firing rates that we observed experimentally were implemented via MF and PF synaptic input and variable leak conductances. Thus the networks had both functional and spatial heterogeneity. Moreover, the automated probabilistic nature of network generation allowed us to examine network dynamics in many different network instantiations.

The modeled volume of GCL contained 45 GoCs, each with 4672 electrical compartments (Figure 7D, dendrites and axons omitted for clarity). The size of network was chosen because the number of connections per cell (Figure S6B) and the cumulative distribution of the connection conductances (Figure S6C) were not significantly different from a larger network of 600 × 600 × 80 μm containing 132 GoCs (Figures S6B and S6C; p > 0.05, n = 5), indicating that connectivity was not very scale sensitive above this size. Calculation of the mean connectivity suggests that an individual GoC makes electrical synapses with 10.5 ± 0.6 (mean range: 9.8–11.5, n = 5 network instantiations) other GoCs (Figure S6B). However, this may be an underestimate given that our connectivity statistics are derived from slice experiments where some dendrites may be truncated.

### Simulated Sparse Excitatory Input Evokes Gap Junction-Mediated Surround Inhibition

Sensory input to the network model was mimicked with a brief burst of eight MF inputs (random train of 200 Hz over 10 ms) followed by 50 temporally delayed PF inputs (random train of 350 Hz over 15 ms) that triggered a burst of two spikes followed by a pause in the GoC(s) receiving excitatory synaptic input, similar to in vivo responses to brief tactile stimuli (Holtzman et al., 2006b; Jorntell and Ekerot, 2006; Vos et al., 1999). When only one cell received excitatory input (Figure 7E, red) two to three neighboring GoCs exhibited a clear inhibition in firing rate (yellow cells; n = 3 networks). When these cells recovered from the depression, their firing depressed the excited cell, extending the duration of the pause following the initial burst, as observed experimentally (e.g., Figures 4A and 4B). When 22% of the cells in the network received suprathreshold synaptic input the network exhibited a mosaic of responses (Figure 7F). Directly excited cells (red) were surrounded by approximately twice as many inhibited cells (yellow). Beside cells showing a pure inhibition of firing (yellow cell, “x”), a few cells exhibited a mild excitation before inhibition (yellow cell, “+”) even though they had no excitatory input. However, this behavior was not observed when only a single cell received input (Figure 7E). These cells mostly showed a depression (in ∼90% of the trials), but when their membrane potential was sufficiently near threshold and neighboring cells fired nearly synchronously, the summation of spikelets (see Figure 2) was sufficient to cross threshold producing a small peak followed by a pause in the peristimulus time histogram (PSTH). These simulations suggest that an electrically coupled GoC network can produce a spectrum of different excitatory and inhibitory response patterns to excitatory synaptic input.

### Simulated Sparse Excitatory Input Evokes Transient Local Network Desynchronization

**Figure 8:**
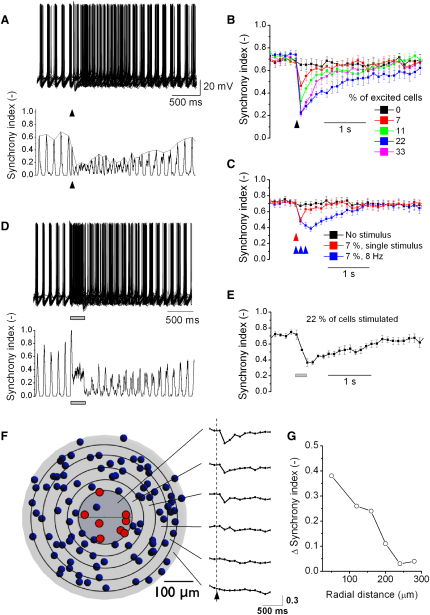
Simulated Sparse Excitatory Inputs Evoke a Transient Local Network Desynchronization (A) (Top) Superimposed spike trains of all 45 Golgi cells (GoCs) in the network. During the baseline period, the GoCs show loose spike synchrony at a population frequency of ∼8 Hz. When ten random GoCs (∼22% of the network) were excited by suprathreshold synaptic input (arrowhead, same input as in Figures 7E and 7F; Experimental Procedures) the GoCs temporarily desynchronized. (Bottom) The temporal evolution of the spike synchrony determined by the synchrony index, SI(t). SI(t) is the total number of spikes within t and t + 20 ms normalized by the number of cells. Thin line connects the rhythmic ∼8 Hz peaks of the SI(t). (B) The dependence of spike desynchronization on the percentage of excited GoCs. After a baseline period, a different number of GoCs received synaptic excitation (arrow). Connected squares show the temporal evolution of the spike synchrony (curves are obtained as illustrated by the thin line in A). Each point (squares) show mean ± SE for ten different randomly connected networks. (C) The dependence of spike desynchronization on the level and duration of the stimulus rate (n = 10). Note that for plots in (B) and (C) the stimulus-evoked spikes were omitted. (D) Example of spike desynchronization during a 250 ms train of asynchronous synaptic input (Experimental Procedures). (E) Mean time course of spike desynchronization (ten networks). (F) (Left) Top view of a cylindrical network (diameter 600 μm, 104 GoCs). Central area is 100 μm in radius and concentric rings are 40 μm wide. Red GoCs received a suprathreshold synaptic input. Right panels show the temporal evolution of the synchrony index following the excitation of the red GoCs (arrow). Traces are averaged over nine network instantiations. (G) Summary of the change in spike synchrony index from the data in (F). See also Figure S7.

In the presence of uncorrelated background synaptic noise, the GoC network model exhibited a mean firing frequency of ∼8 Hz (Figure 8A). We quantified the level of network synchronization by dividing the total number of spikes within a rolling time window of 20 ms by the number of cells. This synchrony index (SI(t)) shows that during background synaptic input the network exhibits loose synchrony with 30–40 of the 45 GoCs firing on each cycle (baseline period in Figure 8A). In contrast, when 10 GoCs (22% of the network) received a temporally precise synaptic input (same as in Figure 7) out of phase with the population rhythm, the network became transiently desynchronized as indicated by the marked reduction in the SI(t) peaks in Figure 8A. Synchronization under quiescent conditions and desynchronization with synchronous excitatory input arose from GJs, because an identical network without electrical coupling exhibited neither spike synchrony before stimulation nor a decrease in synchrony following synaptic stimulation (Figure S7A). The ability of excitatory synaptic input to desynchronize electrically coupled GoC network models was influenced by the value of the GoC density used, but was robust over a 3-fold range (Figure S7B). Moreover, in-phase synaptic input could also desynchronize the network (Figure S7C) albeit to a lesser degree. These results suggest that a brief burst of excitatory synaptic input can transiently switch a GJ coupled GoC network from a synchronized to a desynchronized state.

We explored the properties of network desynchronization by systematically changing the fraction of cells activated by the synchronous synaptic input. As the number of cells excited increased from 3 to 15 (out of a total of 45), the drop in the SI(t) became larger, and the duration of desynchronization increased to several seconds (Figure 8B, n = 10 network instantiations). The reduction in SI(t) was always much larger than expected for a simple phase reset mechanism (e.g., for an 11% excitation and thus phase reset, the GoC network exhibited a 50% reduction in SI[t]). However, when >22% of the GoCs were excited the effect on the SI(t) saturated (Figure 8B). Repeating the sparse synchronous input three times at 8 Hz also increased the level and duration of the network desynchronization (Figure 8C, n = 10). These results suggest that the level of synchrony of an electrically coupled GoC network can reflect the number of excitatory synaptic inputs that have been activated and the extent of their firing.

Beside burst-pause responses to brief tactile stimuli, GoCs can also respond to sensory input with an increase in their firing (up to 50 Hz) for 100–300 ms as observed during joint movement (van Kan et al., 1993), locomotion (Edgley and Lidierth, 1987), and eye saccades (Prsa et al., 2009). To investigate whether this type of response also causes network desynchronization, we mimicked sensory input to the network with eight MF inputs (random train of 200 Hz over 250 ms) followed by 50 temporally delayed PF inputs (random train of 350 Hz over 250 ms) in accordance with in vivo data (Prsa et al., 2009; van Kan et al., 1993). This asynchronous synaptic input to ∼22% of the GoCs in the network transiently increased GoC firing rates to ∼40–50 Hz and also caused a prominent network desynchronization (Figures 8D and 8E, n = 10).

To examine the spatial properties of synaptically evoked network desynchronization, we built a larger network model that consisted of a cylindrical slab of GCL (diameter 600 μm), containing 104 GoCs (Figure 8F). The model was divided in concentric rings. MF and PF inputs were arranged so that they excited eight cells (red) in the central zone in each network instantiation (input as in Figures 8A and 8B). Calculation of SI(t) in each ring across nine different network instantiations revealed the spatial dependence of network desynchronization in response to a local synchronous excitatory synaptic input (Figures 8F and 8G). The peak desynchronization declined steeply over 150 μm and was virtually zero at 250 μm from the center. These simulations suggest that synaptically evoked desynchronization is a local property of the network.

### Determinants of Network Desynchronization

**Figure 9:**
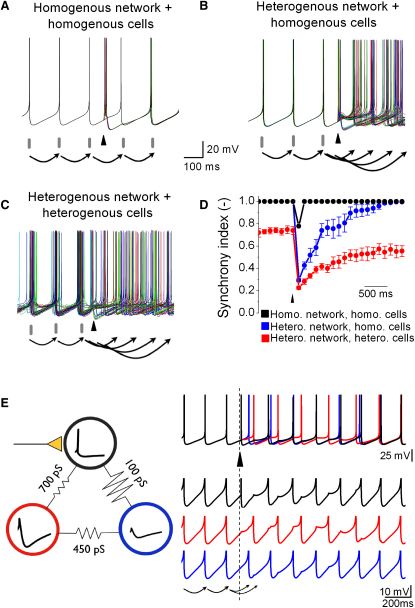
Determinants of Network Desynchronization (A–C) Superimposed voltage traces from 45 Golgi cells (GoCs) in three different network configurations. Ten randomly selected Golgi cells (22%) were excited by supra threshold synaptic input (arrowhead; same as in Figure 8A). (A) Model with spatially homogeneous all-to-all connectivity and GJs of equal conductance between the same location on the ascending dendrites of GoCs with homogeneous cellular properties (each cell has the same input resistance and spontaneous firing rate). (B) Model with spatially heterogeneous connection probability and coupling coefficients as measured (see Figures 7A and 7B), but with homogeneous GoCs. (C) Model with spatially heterogeneous network properties and heterogeneous GoCs (cells have different input resistance and spontaneous firing rates as used in Figures 7 and 8) as observed for real GoCs. (D) Time course of network synchrony after a synaptic input (arrowhead) for the models in (A) (black symbols), (B) (blue symbols), and (C) (red symbols). Data represent mean ± SE. Note that for plots in (D) the stimulus-evoked spikes were omitted. (E) Cartoon of a minimal GoC network showing desynchronization: three identical GoCs coupled together at the soma by electrical synapses with heterogeneous conductances. Only the black cell received synaptic input. (Right) Voltage traces from the black, red, and blue cell. Timing of suprathreshold synaptic input into the black cell indicated by the arrowhead. Spikes truncated in lower three traces for clarity.

Our simulations show that sparse excitatory synaptic input and inhibitory GJ potentials are essential for GoC network desynchronization. To investigate whether other factors are involved in desynchronization, we reduced the complexity of our models to identify the necessary and sufficient components. We first repeated the simulations in Figure 8A using network models with equal and spatially uniform electrical coupling between GoCs with homogenous cellular properties that gave uniform basal firing rates and lacked synaptic background activity (Figure 9A). As expected, this noise-free electrically coupled network became perfectly synchronized in the absence of synaptic input. When 22% of the cells in the network were driven by suprathreshold MF input, the synchronization index dropped by 22%, as expected for a simple phase reset, and then immediately snapped back in phase (Figures 9A and 9D). No desynchronization was observed for this homogeneous network. Moreover, networks with homogeneous connectivity and heterogeneous cellular properties also showed little desynchronization (simulation not shown). In contrast, when network heterogeneity was introduced, MF input strongly desynchronized spiking, even when the cellular properties were homogenous (Figures 9B and 9D). Network heterogeneity introduced by spatial dependences of coupling probability and CC both contributed to network desynchronization. The strongest and longest lasting desynchronization was observed for networks with heterogeneous electrical connectivity and cells with heterogeneity in firing rates and input resistance (Figures 9C and 9D), as observed for real GoC networks. These results show that heterogeneity in electrical coupling is essential for robust network desynchronization while heterogeneous cellular properties can enhance the duration of desynchronization (Figure 9D: red and blue symbols).

To illustrate the mechanisms underlying network desynchronization, we constructed the simplest network model that included the essential components: sparse synaptic input, spontaneously active cells with AHPs, and heterogeneous GJ coupling. The network consisted of three identical GoCs coupled together at the soma by electrical synapses with three different conductances. Only one of the cells received MF input (Figure 9E). The network synchronized in the resting state, but temporally precise synaptic input into the black cell triggered a spike and AHP, that propagated through the GJs to the two other GoCs, inhibiting their firing. Since the spike-phase delay depends on the coupling strength (Figures S3C and S3D), the cell with the weakest coupling (blue) fired before the more strongly coupled red cell (Figure 9E). This spike time dispersion was maintained for more than a second through mutual electrical inhibition. During desynchronization the mean firing rate dropped because inhibition forced cells to skip cycles (black and red cell). Thus, synaptically evoked desynchronization arises in this simple network from heterogeneous GJ coupling strengths, which produce different GJ inhibitory potentials and spike-phase delays, thereby causing temporal dispersion of spike times.

## Discussion

We have investigated the molecular, anatomical, and physiological properties of an electrically coupled interneuron network within the input layer of the cerebellum. We show that Cx36 is necessary for the formation of electrical coupling between GoCs and that the GJs are formed predominantly between apical dendrites in the molecular layer. Paired whole-cell recordings show that coincident excitatory synaptic input can produce both excitatory responses and inhibitory pauses in GoC firing. Pauses in firing were caused by inhibitory GJ potentials, which arise from the efficient propagation of AHPs through GJs to surrounding cells. Inhibitory GJ potentials also caused spike-phase delays and desynchronization of spontaneous firing between cells. These properties were reproduced in detailed GoC network models that incorporated the measured spatial dependence of coupling probability and strength. These models suggest that each GoC is electrically connected to ∼10 others. Network simulations demonstrate that sparse excitatory synaptic input, spike AHPs, and heterogeneous GJ coupling are all essential for triggering network desynchronization. Moreover, they predict that synaptically evoked spike desynchronization is local and that the extent and duration of desynchronization reflects the pattern of synaptic excitation.

### Properties of Electrical Inhibition

Our results show that a synaptically evoked AP in a GoC produces a pause in the spontaneous firing of the surrounding GoCs even though no inhibitory chemical synapses are present between GoCs. GJ coupling therefore converts an excitatory synaptic signal into surround inhibition. GJ potentials exhibit a wide range of waveforms across different cell types (Connors and Long, 2004). Those with a pronounced depolarizing component have an excitatory effect on neighboring cells, promoting lateral excitation (Furshpan and Potter, 1957; Schoppa and Westbrook, 2002; Veruki and Hartveit, 2002) and synchrony detection (Galarreta and Hestrin, 2001b) in response to excitatory sensory stimuli. In contrast, GJ potentials in GoCs have only a small and brief depolarizing component and a large and slow hyperpolarizing component (Dugue et al., 2009). This is caused by the preferential propagation of the slow (∼100 ms) AHP, rather than the brief (∼0.4 ms) AP through the electrical synapses (Figure 2A). The inhibitory action of GJ potentials could be a widespread phenomenon since interneurons with brief APs and large AHPs are found in both cortical and subcortical areas (Galarreta et al., 2004; Galarreta and Hestrin, 1999; Gibson et al., 1999; Hughes et al., 2004; Landisman et al., 2002; Long et al., 2005; Mann-Metzer and Yarom, 1999; Tamas et al., 2000), and it was previously shown in fast-spiking parvalbumin-positive interneurons that a train of presynaptic action potentials could cause a postsynaptic hyperpolarization (Galarreta and Hestrin, 2002).

Besides the AP and AHP shape, several other factors will affect whether the net effect of a GJ potential is inhibitory or excitatory. Neurons where the AP undergoes strong low-pass filtering will favor inhibition. This should be particularly pronounced for neurons that have electrical synapses on their dendrites and do not support active AP back-propagation (Hu et al., 2010). In addition, the membrane potential of the presynaptic cell will determine the driving force for K^+^ and therefore the amplitude of the AHP. Thus, APs evoked from hyperpolarized potentials may evoke GJ potentials with little or no inhibitory component and their effect will be mostly excitatory. Whether a GJ potential is excitatory or inhibitory may therefore be state dependent in some cells. Indeed, cortical interneurons that have been shown to effectively entrain electrically coupled neurons at gamma frequencies (Tamas et al., 2000) may also exhibit surround inhibition at more depolarized potentials.

### Synchronization and Desynchronization of Electrically Coupled Networks

Our results together with those of Dugue et al. (2009) show that GoC networks exhibit spike synchronization in the absence of correlated input and that this is stable even in the presence of significant levels of synaptic noise and heterogeneity in intrinsic firing rates. However, both our paired recording and network simulations demonstrate that sparse excitatory synaptic input can transiently desynchronize the GoC network. These results confirm previous theoretical work, which predicted that pairs of electrically coupled neurons can spike out-of-phase under certain conditions (Chow and Kopell, 2000; Lewis and Rinzel, 2003; Sharp et al., 1992). Moreover, recent theoretical work using an electrically coupled integrate-and-fire (I&F) network with all-to-all homogenous connectivity (Ostojic et al., 2009) predicts that GJ potential shape is an important determinant of network dynamics. While both excitatory and inhibitory GJ potentials could both synchronize the firing, networks with inhibitory GJ potentials were more prone to exhibit asynchronous spiking behavior. Moreover, I&F networks with inhibitory GJ potentials could toggle between stable synchronous or asynchronous states upon external stimulation (Ostojic et al., 2009). Our experimental results and simulations with detailed network models that included spatial dependences of connection probability and coupling strength, and heterogeneity in firing rate and input resistance, show that GoC networks do not exhibit such strict bistability, but instead respond to sparse synaptic stimulation with a transient desynchronization that relaxes back to a default synchronous state.

Sparse synaptic input desynchronizes GoC networks because activation of the innervated cells inhibits neighboring cells, introducing phase delays in their spike times (Figure 9B). Spike-phase delays are different in each cell because the strength of electrical coupling is variable. Once the delayed spikes occur, they, in turn, inhibit their neighbors, maintaining the dispersion of spike times. The amplitude and duration of network desynchronization depends on the fraction of cells in the network that are synaptically activated. However, this effect saturates because the number of noninnervated cells, which drive the spike dispersion, falls and becomes limiting (Figure 8B). During desynchronization, the mean firing rate across the network drops because in those cells that skip cycles, the frequency falls by 25%–50%. The transient nature of network desynchronization can therefore be understood in terms of a perturbation of the mean firing rate away from the natural firing frequency (∼8 Hz) of these loosely coupled oscillators. Moreover, previous studies have shown that the conditions for network synchrony and asynchrony and their stability depend on intrinsic membrane conductances and firing frequency (Mancilla et al., 2007; Pfeuty et al., 2003). Although these studies focused on steady-state network behavior in the absence of external synaptic input, their findings imply that the duration of the transient desynchronization we observe may be interneuron-type specific.

Network oscillations are also known to be dependent on the neuromodulatory state of the network (Fisahn et al., 1998), but desynchronization often appears to be too fast to be mediated by changes in neuromodulation. Previous studies have suggested that GABAergic inhibition in the thalamic reticular nucleus is involved in thalamocortical desynchronization (Huntsman et al., 1999; Sohal et al., 2000). Interestingly, nerve cells in this nucleus show strong electrical coupling and large AHPs (Landisman et al., 2002) raising the possibility that electrical synapses could also be involved in desynchronization.

### Potential Mechanisms Underlying the Diverse Behavior of Golgi Cells In Vivo

In vivo recordings show that GoCs are tonically active and respond to sensory input in a variety of ways. This depends on the both the properties and modality of the sensory input. MF inputs can convey graded information such as joint angle (van Kan et al., 1993) and head velocity (Arenz et al., 2008; Barmack and Yakhnitsa, 2008) with a modest and relatively slow bidirectional modulation of their firing rate (Barmack and Yakhnitsa, 2008; Edgley and Lidierth, 1987). GoCs are also likely to respond to these inputs in a graded bidirectional way. In contrast, GoC responses to discrete tactile stimulation of whiskers, face, and limbs is quite different, evoking either a spike-burst followed by a pause in firing (Holtzman et al., 2006b; Jorntell and Ekerot, 2006; Vos et al., 1999), or more commonly, a characteristic inhibitory pause in firing (Holtzman et al., 2006a, 2006b). While the excitatory responses can potentially be explained by feed-forward excitation by MFs and feedback excitation by PFs both the subsequent pause and the regular occurrence of purely inhibitory, 30–500 ms pause-only responses (Holtzman et al., 2006b) have been more difficult to explain.

Several mechanisms have been proposed to underlie sensory-evoked inhibitory pauses in GoC firing (discussed in Holtzman et al., 2006a, 2006b; Xu and Edgley, 2008). Our experimental results argue against inhibitory input being a major determinant of pauses and show that sparse synaptic excitation and inhibitory GJ potentials can account for both the excitation-pause and pause-only responses observed in vivo. Moreover, network simulations predict that MF stimulation causes two to three times more inhibitory GoC responses than excitatory (Figures 7E and 7F), consistent with in vivo data showing that GoC pauses are more common (Holtzman et al., 2006b). Xu and Edgley (2008) recently showed that climbing fiber (CF) stimulation can also cause GoC pauses. Our results suggest that inhibition mediated by GJs could underlie this observation if CFs directly innervate GoCs, but data supporting CF- GoCs synapses are scarce. Alternatively, CF stimulation might inhibit GoCs through Lugaro cells, molecular layer interneurons, or Purkinje cells. Although our data suggest that disynaptic inhibition is weak, we do not rule out the possibility that such chemical inhibitory pathways could contribute to the GoCs pauses observed in vivo.

### Potential Role of Synchronization and Desynchronization in the Cerebellar Granule Cell Layer

Local field potential (LFP) oscillations in the theta band (rodents, ∼8 Hz) (Dugue et al., 2009; Hartmann and Bower, 1998; O'Connor et al., 2002; Ros et al., 2009) and beta band (monkeys, 13–18 Hz) (Courtemanche and Lamarre, 2005; Courtemanche et al., 2002; Pellerin and Lamarre, 1997) are observed in the GCL in vivo. These oscillations can be phase-locked with LFPs in the primary somatosensory (Courtemanche and Lamarre, 2005; O'Connor et al., 2002; Ros et al., 2009) and motor cortices (Courtemanche and Lamarre, 2005) and are likely to be involved in preparing the motor system for the execution of movements (Courtemanche and Lamarre, 2005; Hartmann and Bower, 1998). Indeed, in both rodents and monkeys, these GCL oscillations are correlated most clearly with behaviors described as “quiet wakefulness” and “expecting” but usually disappear rapidly upon movement (Courtemanche et al., 2002; Hartmann and Bower, 1998; Pellerin and Lamarre, 1997).

At present, the function of spike desynchronization and disappearance of oscillations in the cerebellar GCL is unclear. Golgi cells can inhibit thousands of granule cells and every granule cell receives inhibitory input from ∼4–8 GoCs (Jakab and Hamori, 1988; Rossi and Hamann, 1998). The downstream effect of GoC synchronization at theta frequency will be to coordinate phasic inhibition onto granule cells, producing rapid pulses of inhibitory conductance, and possibly slower waves mediated by spillover- and tonic inhibition (Crowley et al., 2009; Dugue et al., 2009; Kanichay and Silver, 2008; Rossi and Hamann, 1998). A theoretical study has predicted that this rhythmic inhibition will introduce permissive and nonpermissive time windows for synaptic integration at 10 Hz and that MF input applied during asynchronous inhibition will be less likely to trigger spikes (Kistler and De Zeeuw, 2003). This conclusion is consistent with theoretical work showing that inhibitory input synchrony increases the gain in neocortical neuron models (Tiesinga et al., 2004), but the downstream effects on network excitability are difficult to predict (Schroeder and Lakatos, 2009) without a full network model. Nevertheless, variations in inhibitory input synchrony may be a dynamic way to alter granule cell gain during sensory input, in addition to the steady-state tonic inhibitory component (Crowley et al., 2009; Mitchell and Silver, 2003; Rothman et al., 2009). Finally, the transient nature of network desynchronization could allow the cerebellar input layer to act as a timing device over the 10 ms to 1 s timescale (Medina et al., 2000) or as a short-term-memory storage mechanism (Ostojic et al., 2009).

Our results show that sparse synaptic excitation can desynchronize tonically active interneuron networks by triggering GJ-mediated surround inhibition. If local networks of interneurons with inhibitory GJ potentials are innervated by sparse long-range excitatory connections, electrical inhibition could be used to trigger near-simultaneous desynchronization of multiple networks across the brain.

## Experimental Procedures

For full methodological details see Supplemental Experimental Procedures.

### Slice Preparation and Electrophysiology

Sagittal slices of cerebellar vermis were prepared from mice (P13–P37) in accordance with UK Home Office guidelines. Electrophysiological recordings were carried out in ACSF at 35–38°C. GoCs were identified and MF and PF stimulation was performed as previously described (Kanichay and Silver, 2008).

### Neurolucida Reconstructions and Electron Microscopy

After recordings, biocytin was visualized using avidin-biotin-horseradish peroxidase complex. Sections were processed as previously described (Biro et al., 2005; Golding et al., 2005) and reconstructions performed with a Neurolucida system. Close appositions between the processes of the cell pairs were checked with ultrathin 70 nm sections in the EM (Tamas et al., 2000).

### Immunohistochemistry

Sections of cerebellar vermis from male P45 Wistar rats and P16 mice were processed and incubated in rabbit polyclonal anti-mGluR2/3 (Chemicon, Temecula, CA) and mouse monoclonal anti-Cx36 (Chemicon). Immunoreactions were visualized with Alexa488 conjugated goat anti-rabbit and goat anti-mouse (Molecular Probes) and Cy3-conjugated goat anti-mouse (Jackson ImmunoResearch) and imaged with a confocal microscope. Single images or maximum intensity Z-projection images (6–15 images) are presented.

### Data Acquisition and Analysis

Recordings were digitally filtered at 7 kHz and analyzed with Neuromatic and Origin 8 (OriginLab). Cells were regarded coupled when the CC was >1%. Pooled data are expressed as mean ± SE unless stated otherwise. Sample means were compared with a two-sided Wilcoxon signed rank test and considered significant at p < 0.05. Phase-response curves and cross-correlograms were calculated as previously described (Dugue et al., 2009).

### Golgi Cell Model and Network Simulations

GoC pair and network models were build using neuroConstruct (Neuroconstuct.org; Gleeson et al., 2007) using our reconstructed GoC morphologies and active conductances from a previous model (Solinas et al., 2007). Simulations were performed with NEURON (Carnevale and Hines, 2006). MF and PF synaptic conductances were based on measured EPSCs (Dieudonne, 1998; Kanichay and Silver, 2008). Electrical coupling was determined stochastically for each network instantiation. Simulation time steps were 0.025 and 0.05 ms for the GoC pair and network models, respectively. Models available from neuroConstruct.org, NeuroML.org, and the ModelDB database.

## Supplemental Information

Document S1. Experimental Procedures, Figures, and Table
