# Supplementary material for: Rapid Desynchronization of an Electrically Coupled Interneuron Network with Sparse Excitatory Synaptic Input
Source: Neuron. 2010 Aug 12;67(3-2):435–51. doi: 10.1016/j.neuron.2010.06.028 (PMC2954316; doi:10.1016/j.neuron.2010.06.028)
Supplement: Document S1. Experimental Procedures, Figures, and Table [file mmc1.pdf]

Supplemental Information   Neuron, Volume 67

Rapid desynchronization of an electrically coupled interneuron network with sparse excitatory synaptic input

Koen Vervaeke, Andrea Lőrincz, Padraig Gleeson, Matteo Farinella, Zoltan Nusser &

R. Angus Silver.

Inventory

1. Supplemental Figures 2

Figure S1 (Related to Figure 1) 2

Figure S2 (Related to Figure 2) 3

Figure S3 (Related to Figure 4) 4

Figure S4 (Related to Figure 5) 5

Figure S5 (Related to Figure 6) 6

Figure S6 (Related to Figure 7) 7

Figure S7 (Related to Figure 8) 8

2. Supplemental Table S1 (Related to Figure 6) 9

3. Supplemental Experimental Procedures 11

4. Supplemental References 17

1. Supplemental Figures

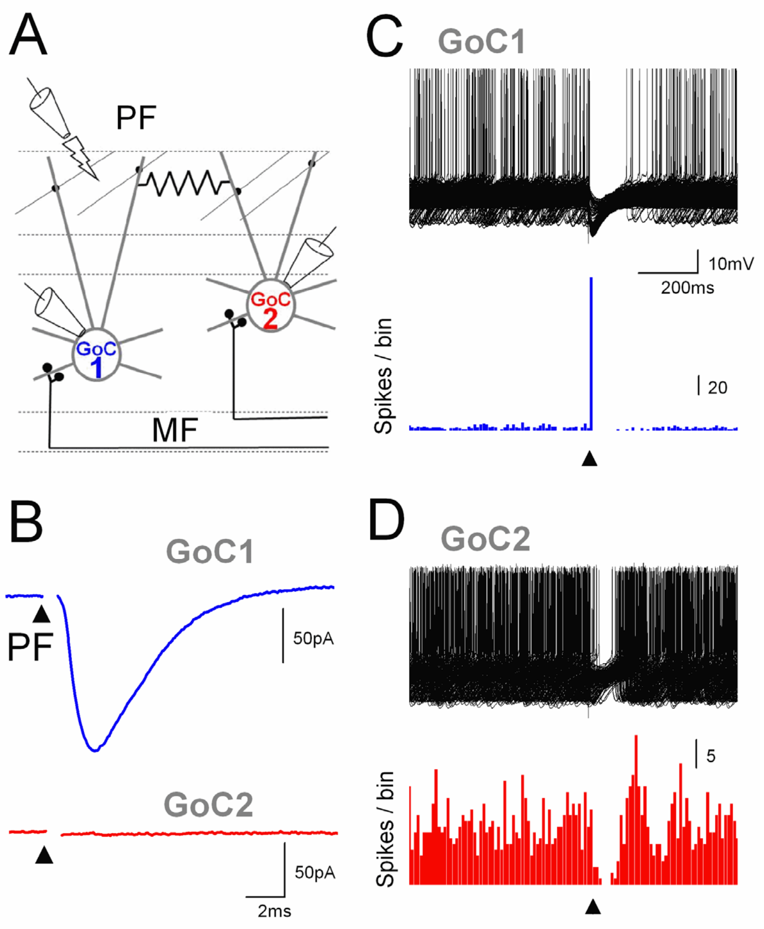

Figure S1 (Related to Figure 1)

Fig.S1. Parallel fiber stimulation can excite or inhibit Golgi cells. **(A)** Schematic diagram of paired Golgi cell (GoC) recording configuration with parallel fiber (PF) stimulation (MF; mossy fiber input). The coupling coefficient (CC) for this pair was 15.0 %. **(B)** PF Stimulation produced an EPSC only in GoC1 (V_{hold} = -70 mV) in the presence of the 10 μM gabazine and 0.5 μM strychnine to block inhibition. **(C)** Superimposed voltage recordings showing firing of GoC1 during single shock PF stimulation (same stimuli as in (B)), which reliably evoked a spike followed by a pause in firing. Bottom panel shows spike histograms (10 ms bins). (**D**) Superimposed voltage recordings showing firing of GoC2 during the same trials as (C), together with spike histograms below. GoC2 responded to PF input into GoC1 with an inhibitory pause.

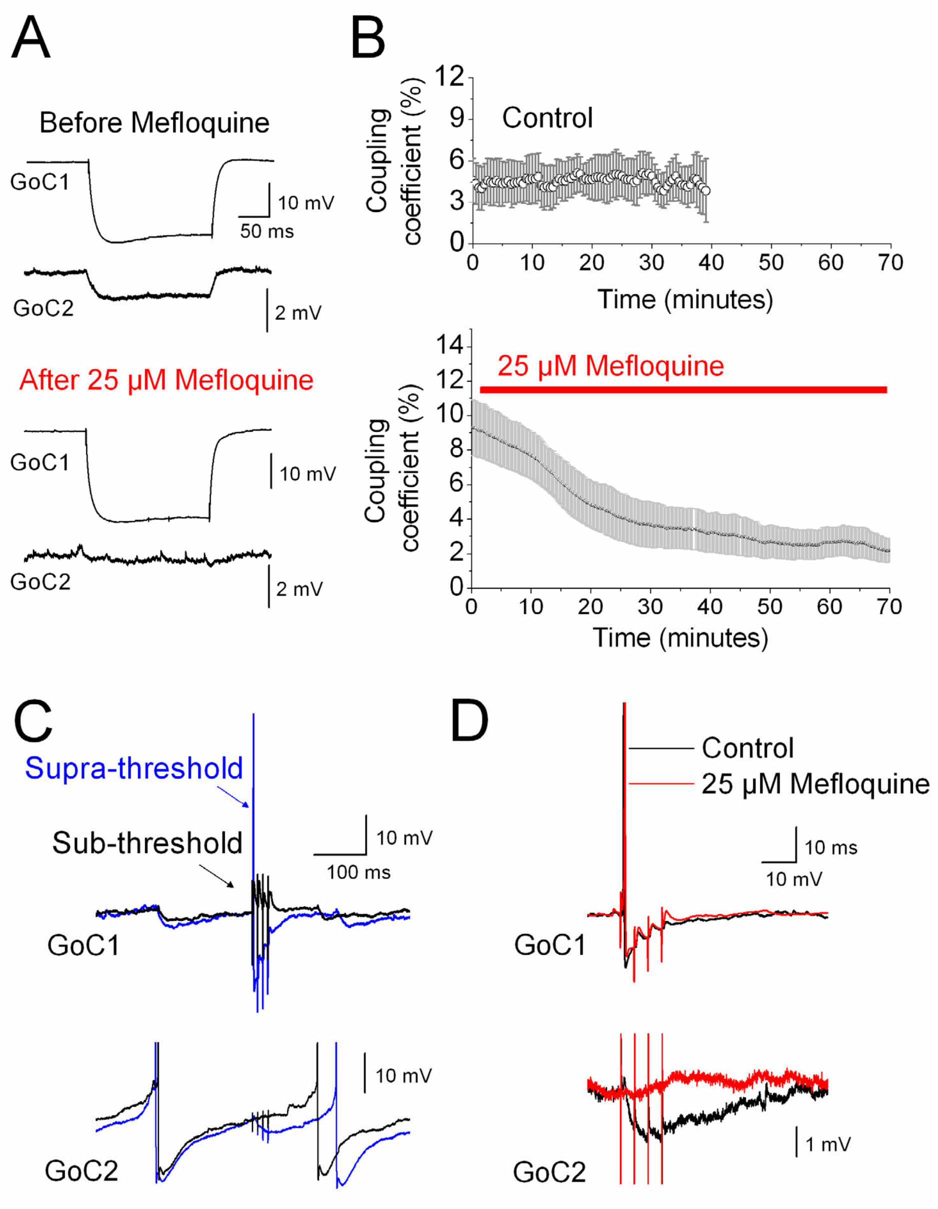

Figure S2 (Related to Figure 2)

Fig.S2. Electrical coupling between Golgi cells and inhibitory gap junction potentials are sensitive to mefloquine. **(A)** Voltage traces of electrically coupled Golgi cells (GoC) while injecting a -0.2 nA current pulse in GoC1, before (top traces) and after (bottom traces) bath application of 25 μM mefloquine. **(B)** Top: Time course of the coupling coefficient (Mean ± SE) when no drugs are applied (n = 4 pairs). Bottom: Time course of the effect of bath application of 25 μM mefloquine on the coupling coefficient (Mean ± SE, n = 5 pairs). Note that the coupling coefficient was stable with time under control conditions while mefloquine application strongly reduced the coupling coefficient. **(C)** Voltage traces of an electrically coupled GoC pair while stimulating mossy fiber (MF) input into one of the cells (4 stimuli at 100 Hz). Black traces: MF stimulation is just subthreshold in GoC1 and causes no hyperpolarization in GoC2. Blue traces: MF stimulation triggers a spike in GoC1 which causes a hyperpolarization in GoC2. Steady current in GoC1 was used to adjust the spike probability. **(D)** Voltage traces of an electrically coupled GoC pair while stimulating MF input into one of the cells (4 stimuli at 100 Hz) before and after mefloquine application. After application of 25 μM mefloquine, the hyperpolarization in the postsynaptic cell disappeared indicating that Cx36 containing gap junctions are responsible for the inhibitory gap junction potential. These data indicate that the spike AHP causes the inhibition in GoC2.

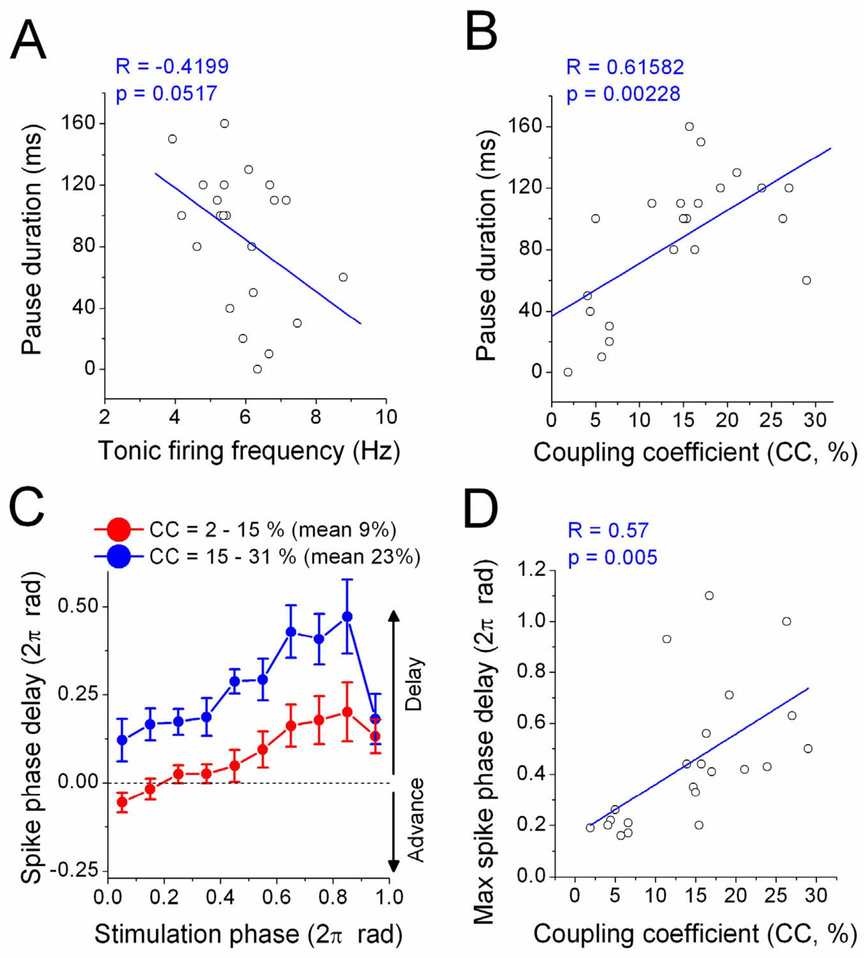

Figure S3 (Related to Figure 4)

Fig.S3. Dependence of Golgi cell pause duration and spike phase delay on Golgi cell tonic firing frequency and coupling coefficient (CC)**. (A)** Relation between the Golgi cell pause duration and its tonic firing frequency. Higher tonic firing frequencies tended to have shorter pause durations. All experiments were included (see example in Fig.4A) regardless of the CC or whether pauses in firing were evoked with parallel fiber or mossy fiber stimulation (n = 22). **(B)** Relation between the pause duration and CC. Lower CC resulted in shorter pause durations (same data set as in (A)). **(C)** Phase response curves (PRC) for two different levels of electrical coupling (low coupling: red, CC: 2-15% mean 9% n = 11; high coupling: blue, CC: 15-31% mean 23% n = 11). Low electrical coupling (red) resulted in a smaller spike delay than high coupling (blue). All experiments are included regardless tonic firing frequency or whether depressions were evoked with parallel fiber or mossy fiber stimulation. Circles present mean ± SE. Data was binned in phase bins of 0.1 (* 2π radians). Both groups were significantly different ($p < 0.05$) for all phase bins, except the ones centered on phase 0.85 ($p = 0.056$) and 0.95 ($p = 0.57$). **(D)** Maximum spike phase delay as a function of CC (same data as in (C)).

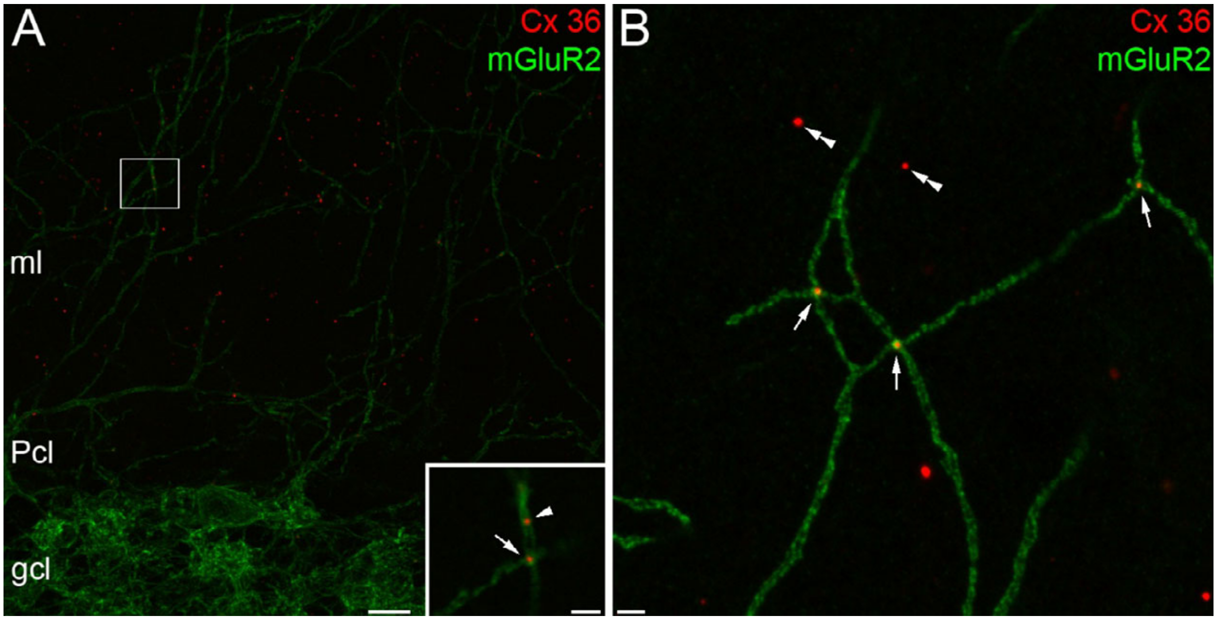

Figure S4 (Related to Figure 5)

Fig.S4. Molecular identity and location of gap junctions between Golgi cells in rats. **(A-B**) Double immunofluorescent reactions for connexin 36 (Cx36) (red) and mGluR2 (green) in rat cerebellar cortex. Some Cx36 immunoreactive puncta were detected in the dendrites of mGluR2 immunopositive Golgi cells (arrowhead) and often localized at the intersections (arrows) of two mGluR2 immunopositive dendrites originating from different Golgi cells. The majority of the intense Cx36 immunopositive puncta were not associated with mGluR2 immunopositive dendrites (double arrowheads). Inset in (A) is a high magnification view of the boxed area. Images are maximum intensity Z projections of (A and B: 15, A inset: 6) confocal sections (A inset, B: 0.5 μm, A:1 μm separation). Scale bars, A: 10 μm; A inset, B: 2 μm.

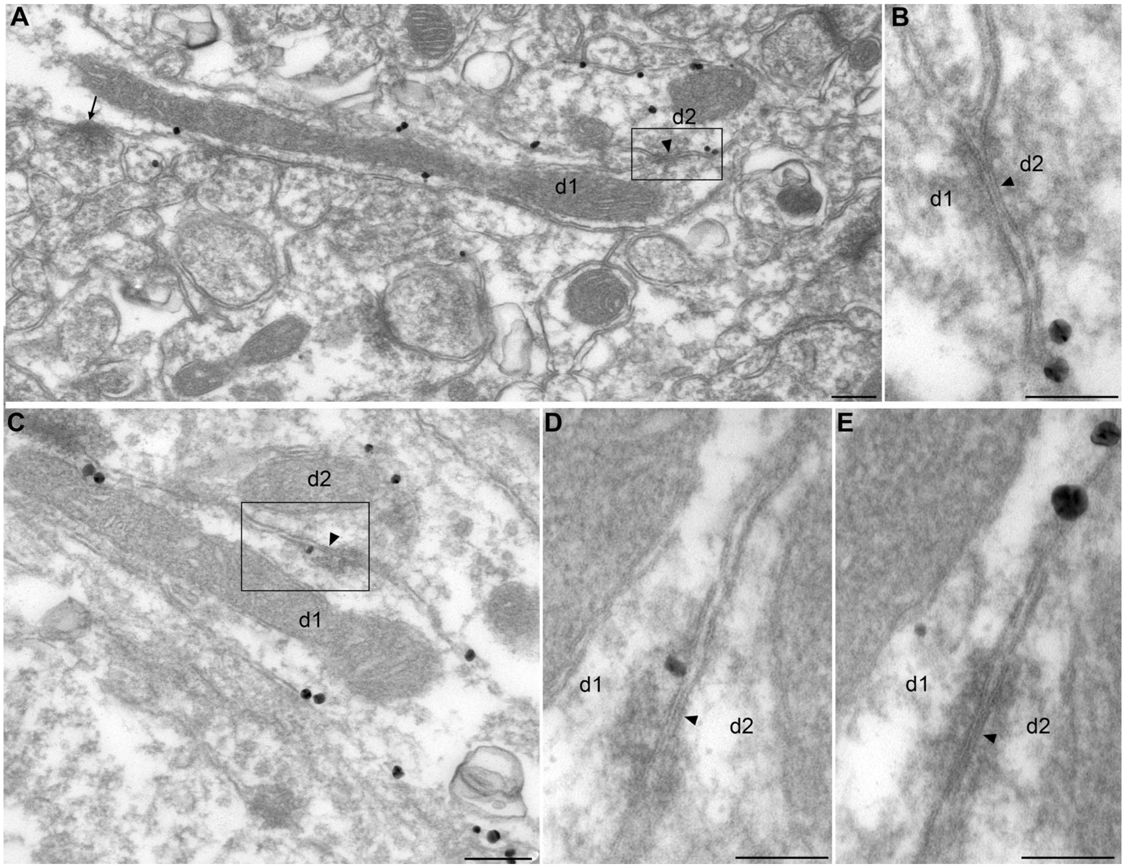

Figure S5 (Related to Figure 6)

Fig.S5. Electron microscopic localization of gap junctions between mGluR2 immunopositive Golgi cell dendrites in mouse (P23) cerebellar cortex. **(A)** Low magnification image of two Golgi cell dendrites (*d1* and *d2*) coupled by a gap junction (arrowhead) in the molecular layer. Golgi cell dendrites were identified by the plasma membrane-associated immunogold particles labeling mGluR2. The arrow indicates an excitatory synapse formed on *d2*. **(B)** High magnification view of the boxed area shown in (A) demonstrates the presence of a gap junction (arrowhead) between *d1* and *d2*. **(C)** Low magnification image shows another example of two mGluR2 immunopositive Golgi cell dendrites (*d1* and *d2*) connected with a gap junction (arrowhead). **(D-E)** The ultrastuctural features of the gap junction (arrowhead) between *d1* and *d2* are shown at high magnifications on the same (D; boxed area in C) and on a consecutive section (E). Scale bars: A, C: 200 nm; B, D and E: 100 nm.

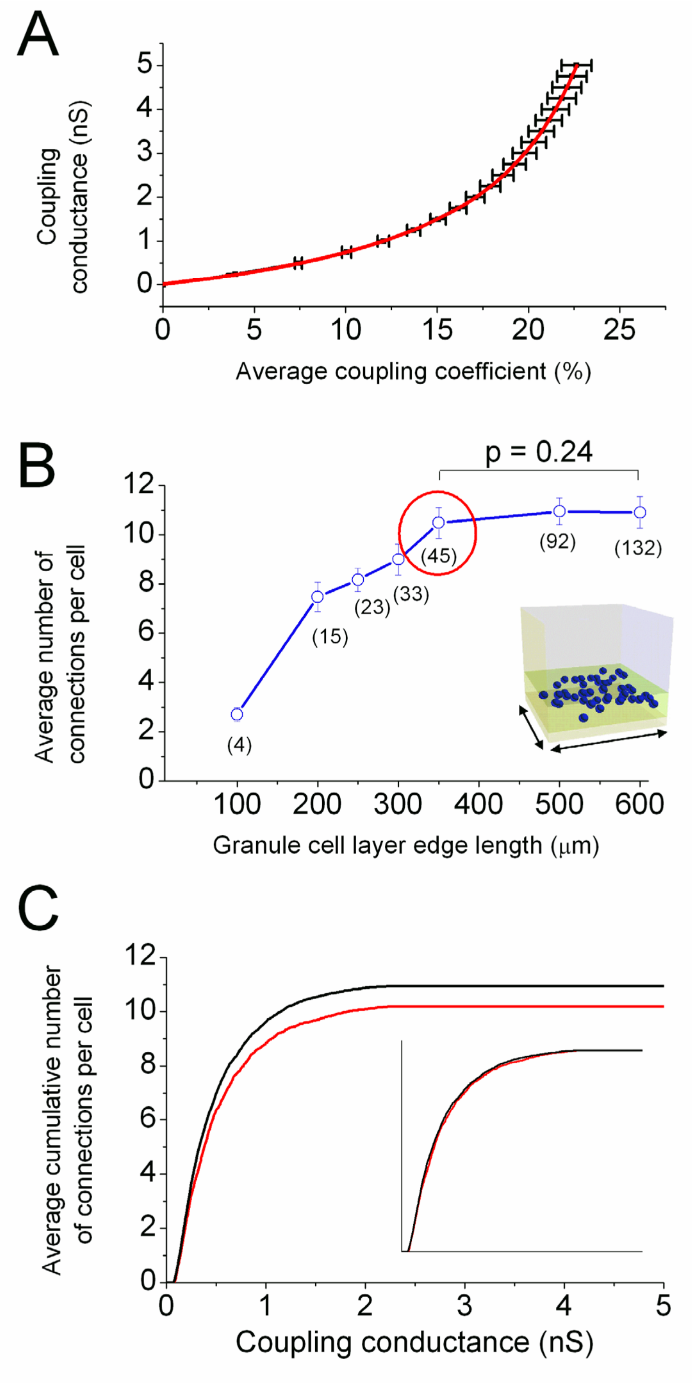

Figure S6 (Related to Figure 7)

Fig.S6. Connectivity statistics for the Golgi cell network models. **(A)** The average relation between coupling conductance and coupling coefficient obtained empirically with simulations using the 2-cell model. **(B)** Average number of electrical connections per cell as a function of the edge length of the granule cell layer (GCL; both equal, arrows). The thickness was fixed to 80 µm as measured experimentally. Average values were obtained from 5 network instantiations. The number of cells for each model size is indicated in parentheses. The red circle indicates the size of the model used for all the simulations unless indicated otherwise. The average number of connections per Golgi cell in the larger model (600 µm edge length) was not significantly larger than in the model used for simulations. **(C)** Average cumulative distribution of coupling conductances between Golgi cells in the larger model (black) and the model used for the simulations (red). When normalized, these distributions overlap as shown in the inset. Data from 5 network instantiations.

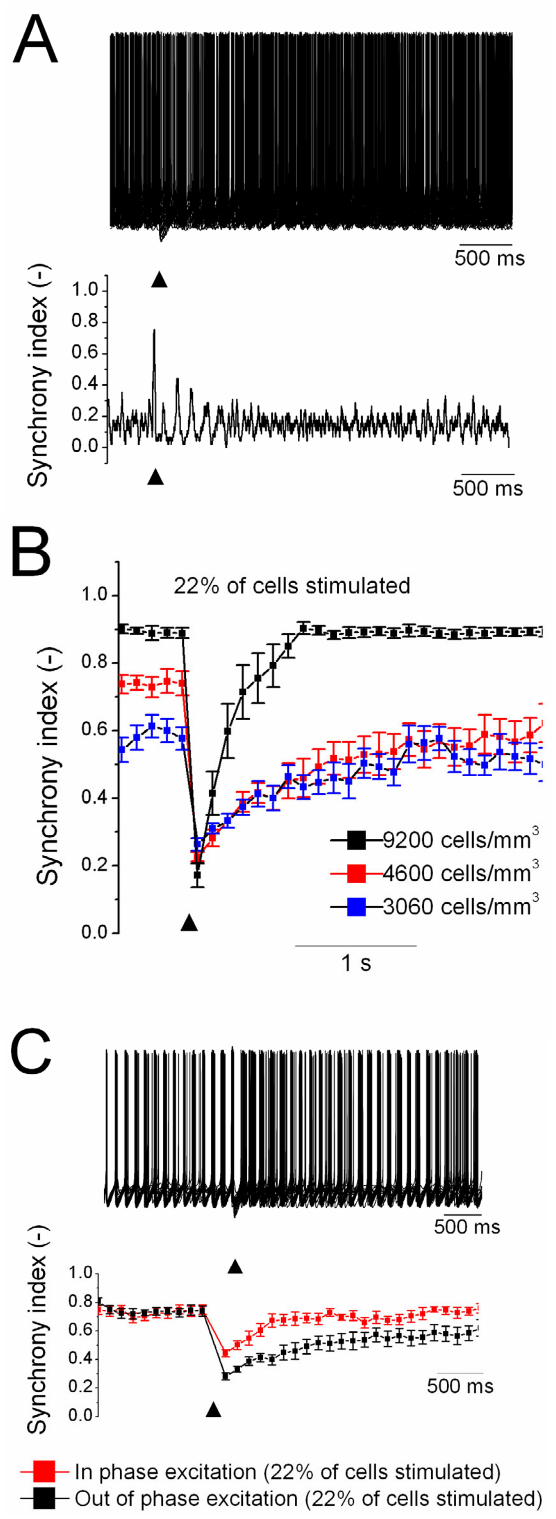

Figure S7 (Related to Figure 8)

Fig.S7. Effect of sparse excitatory synaptic input on network synchrony under various conditions. **(A)** Network model without gap junctions. Top: overlapping voltage traces of all 45 Golgi cells (GoCs) in the network. Bottom: the temporal evolution of the spike synchrony was determined by the synchrony index, SI(t). During the baseline (before arrow head) the cells showed no spike synchrony (top) and thus no clear peaks in the synchrony Index (SI(t), bottom). When 15 out of 45 randomly selected cells (~33 % of the network) received suprathreshold synaptic input (arrow head) the spike synchrony temporarily increased as indicated by a transient appearance of peaks in the SI(t) (bottom). Note that during synaptic excitation (arrow head), SI(t) increases transiently to ~0.8 because most cells receiving synaptic input respond with spike doublets. (**B)** Comparison of the spike desynchronization in network models with different Golgi cell densities (n = 10 network instantiations for each case). Red symbols represent the standard GoC density used for the simulations in Fig.7, Fig.8 and Fig.9. **(C)** Comparison of the spike desynchronization for a standard network configuration (see main text) when cells were excited with temporally precise mossy- and parallel fiber input either in-phase or out-of-phase with the population firing (n = 10 network instantiations).

2. Supplemental Table S1 (Related to Figure 6)

NA = Not Applicable

Parameter	Experiment		Golgi Cell Model		Golgi Cell Model (Solinas et al. 2008)
	Mean ± SE	Range			
Input Resistance[1,*] (MΩ)	179 ± 4 (n=230)	74 - 431	210	NA	245
Cell Capacitance[2,*] (pF)	64 ± 3 (n=44)	32 - 105	79.5	NA	97
f/I relation[3,*] (Hz/pA)	0.25 ± 0.02 (n=9)	0.15 - 0.35	0.18	NA	0.18
Spike Width[4,*] (ms)	0.43 ± 0.03 (n=9)	0.36 - 0.60	0.4	NA	0.39
Spike Threshold[5] (mV)	-40.9 ± 0.5 (n=62)	-50 - -33	-41.5	NA	41
Spike Amplitude[6] (mV)	63.5 ± 4.8 (n=12)	54 - 78	86.8	NA	76
Spike AHP amplitude[7] (mV)	23.1 ± 0.4 (n=62)	15 - 33	21	NA	23.9
			Mean ± SE	Range	
Spikelet amplitude[8,*,†] (mV)	0.49 ± 0.11 (n=9)	0.22 - 1.2	0.66 ± 0.06 (n=10)[‡]	0.45 - 1.0	NA
Spikelet width[9,*,†] (ms)	5.5 ± 0.18 (n=9)	4.9 - 6.3	6.9 ± 0.07 (n=10)[‡]	6.6 – 7.3	NA
Spikelet AHP amplitude[10,*,†] (mV)	0.94 ± 0.12 (n=9)	0.3 - 1.37	1.06 ± 0.02 (n=10)[‡]	1.0 - 1.1	NA

* Experimentally, when cells were spontaneously active, negative steady current was injected to stop spiking (typically -5 to -100 pA). In the models, a -15 pA steady current was added to the soma to silence spontaneous firing. The membrane potential in models and experiments varied between -55 and -60 mV.

† In order to compare model and experiments, the coupling strength between model cells was adjusted to match the average coupling strength in the experiments (model: CC = 12.7 ± 0.24 %, experiment: CC = 13.2 ± 1.82 %)

‡ For every simulation, two reconstructed Golgi cell models were randomly connected with an electrical synapse.

[1] Input Resistance (R_input) was calculated by Ohms law from the steady state voltage response to a 200 ms, -100 pA pulse.

[2] Cell capacitance was determined by fitting a mono exponential function to the first 40 ms of the voltage response to a 200 ms, -100 pA pulse. The capacitance was then calculated from C = Tau / R_input, with Tau being the time constant of the exponential fit.

[3] The average spike frequency was calculated in response to a 200 ms current pulse of 0, 100 and 200 pA. The slope was subsequently determined from a linear fit.

[4] The width was determined at the half maximum spike amplitude (see [6]).

[5] The spike threshold was determined where the dV/dt of the spike was larger then 5 mV/ms

[6] The spike amplitude was determined from the spike threshold to the spike peak.

[7] The spike AHP amplitude was determined from the AHP minimum to the spike threshold.

[8] The spikelet amplitude was determined from the baseline to the peak.

[9] Spikelet width was determined at the base of the spikelet.

[10] Spikelet AHP was determined from baseline to the spikelet AHP minimum.

3. Supplemental Experimental Procedures

Slice preparation and electrophysiology

Experiments complied with U.K. Home Office guidelines. For most experiments C57BL/6 mice (age: P13 – P28, mean: P17) were used. There was no correlation between age and coupling strength ($p > 0.05$). $Cx36^{-/-}$ and $Cx36^{+/+}$ (Deans et al., 2001) littermates (age: P14-P37, mean: P23) were used for data in Fig.5. Mice were anaesthetized with isoflurane and saggital vermal cerebellar slices (280 µm) were prepared as previously reported for rats (Kanichay and Silver, 2008) except for the slicing solution, which contained in mM: 87 NaCl, 2.5 KCl, 7 $MgCl_2$, 1.25 NaH_2PO_4, 25 $NaHCO_3$, 25 glucose, 75 sucrose, 1 kynurenic acid, bubbled with 95% O_2/5% CO_2. Slices were either stored at room temperature or kept at 32 °C for up to ~6 hours before use. Experiments were carried out in ACSF, which contained in mM: 125 NaCl, 2.5 KCl, 2 $CaCl_2$, 1 $MgCl_2$, 1.25 NaH_2PO_4, 26 $NaHCO_3$, and 25 glucose, pH 7.3, equilibrated with 5%CO_2 / 95%O_2 at 35-38°C (< 0.5 °C change in a single experiment). Patch pipettes (4 – 8 MΩ, (Kanichay and Silver, 2008)) contained internal solution: In mM: 150 $KMeSO_4$, 6 NaCl, 1 $MgCl_2$, 0.03 EGTA, 10 HEPES, 4 ATP-Mg, and 0.4 GTP-Na_2, titrated to pH 7.35 with KOH (Dugue et al., 2009) with 0.1% w/v biocytin, except for voltage clamp of spikelets (Fig 5.G,H) and measurement of mossy fiber stimulation evoked IPSCs, which contained In mM: CsGluconate 120, HEPES 10, EGTA 5, GTP 0.5, ATP 4, QX-314(Cl) 5, TEA(Cl) 10, Biocytin 0.1 %. pH adjusted to 7.3 with CsOH.

Current-clamp and voltage-clamp recordings were performed with a Multiclamp 700B amplifier (Molecular Devices). Data was low-pass filtered at 10 kHz and digitized at 20-50 kHz. Recordings were acquired with Neuromatic (http://www.neuromatic.thinkrandom.com/) running within the IgorPro environment (Wavemetrics). Golgi cells (GoCs) were selected as described in (Kanichay and Silver, 2008). In addition their morphology was assessed by biocytin labeling except in a few cases where Alexa 594 (100 µM; Invitrogen) was added to the internal solution and the cells were visualized with a CCD camera as described previously (Kanichay and Silver, 2008) or with 2-photon imaging. After acquiring I/V and f/I relationships at the beginning of experiments, 10 µM gabazine and 500 nM strychnine were routinely added to the bath to block synaptic inhibition (except where stated otherwise). The GoC membrane time constant was 9.8 ± 2.4 ms (mean ± SD, n = 44). After blocking synaptic inhibition, some GoCs that were silent or

had a very low firing frequency were injected with a steady positive current so their firing frequency reflected that typically observed in mice *in vivo* (2-10 Hz) (Barmack and Yakhnitsa, 2008; Ros et al., 2009). Mossy fibre (MF) and Parallel fibre (PF) stimulation was performed as previously described (Kanichay and Silver, 2008). To test that GoCs were not directly stimulated, EPSCs were visually inspected for latency (> 1.5 ms), shape, and short-term plasticity during a 100 Hz pulse train. Moreover, at the end of the experiment, responses were blocked by 10 μM NBQX and 50 μM APV (Rs < 20 MΩ). For measurements of MF evoked di-synaptic IPSCs Golgi cells were held at the reversal potential for excitatory synaptic input. The IPSC conductance was calculated based on the calculated Cl^- reversal potential (-58 mV) and the maximal NBQX-sensitive IPSC response during 10 pulses at 100 Hz.

The following protocols were used to determine whether Golgi cells were coupled by chemical inhibitory synapses. During paired GoC recordings, before adding gabazine and strychnine, we routinely performed input-output relationships in both cells. Thus spikes were triggered with current pulses in one cell while monitoring the postsynaptic response in the other cell, both in current clamp (~-60 mV) and in voltage clamp (~-40 mV to -50 mV, calculated Cl^- reversal potential: -75 mV). Inhibitory gap junction potentials (GJPs) were distinguished from inhibitory chemical synaptic potentials using the following criteria; 1) The outward component of GJPs has a much slower rise time than IPSPs or IPSCs, 2) GJPs show little voltage dependence compared to IPSPs or IPSCs. 3) GJPs do not show short-term plasticity, which IPSPs or IPSCs often do. 4) Finally, gabazine and strychnine were added to see whether the responses were blocked.

Data acquisition and analysis

Recordings were analyzed with Neuromatic and Origin 8 (OriginLab). All figure traces were digitally filtered at 7 kHz using a binomial smoothing function. The membrane potential was not corrected for the junction potential. To determine input resistance and coupling coefficient (CC) negative current (typically 5 - 100 pA) was applied to silence spontaneous spiking. Input resistance was calculated from a 200 ms, -200 pA pulse. Cells were regarded coupled when the CC was > 1 %. Pooled data are expressed as mean ± SE unless stated otherwise. Sample means were compared with a two sided Wilcoxon signed rank test and considered significant at $P < 0.05$. Phase-response curves and cross-correlograms were calculated as previously described (Dugue et al., 2009). The cross-correlations in Fig.3C,D were calculated as following;

first stimulation was regarded in-phase if the stimulation occurred within ± 0.1 of the cycle around the expected spike time. Only sweeps (~100 - 200) were selected where cells showed 2 consecutive synchronized spikes before stimulation. Second, the peristimulus time histogram (PSTH) of the spike times in a 300 ms time window before stimulation or in a 400 ms time window after stimulation were constructed for both cells (10 ms bins). Finally, both PSTH were filtered with a 3-point adjacent average filter and cross-correlated. The pause duration was defined as the interval between the stimulus and the second PSTH bin after the stimulus that was larger than the mean bin height before the stimulus.

Neurolucida reconstructions and Electron microscopy

After recordings, slices were placed in a fixative containing 4% paraformaldehyde and 1.25% glutaraldehyde in 0.1 M phosphate buffer (PB; pH 7.4) and left for several days. Slices were cryoprotected in 10% and 20% sucrose solutions (in 0.1 M PB) for 45 min followed by freezing and thawing. After several washes in PB, slices were embedded in 1% agarose and re-sectioned at 60 μm thickness. Biocytin was visualized using avidin–biotin–horseradish peroxidase complex. Sections were then dehydrated and embedded in epoxy resin (Durcupan) as described earlier (Biro et al., 2005). The GoC reconstruction used for the network simulations was processed according to (Golding et al., 2005). Three-dimensional light microscopic reconstructions of the cells were performed with the Neurolucida system (MicroBrightField) using a 100x oil-immersion objective. Light micrographs of each close apposition were used for the EM identification of the GJs. 70 nm serial sections were cut with an ultramicrotome. All close appositions between the filled processes of the two cells were checked on the ultrathin sections in the EM (Tamas et al., 2000).

Immunohistochemistry

Adult (P45) male Wistar rats and P16 male mice were deeply anesthetized with ketamine and xylazine. They were perfused through the aorta, first with 0.9 % saline for 1 minute, then with ice-cold fixative containing 4% paraformaldehyde and 15 v/v % picric acid in 0.1 M PB (pH = 7.3) for 10 min. 60 μm thick sections from the cerebellar vermis were cut with a Vibratome (VT1000S, Leica Microsystems) and were washed in 0.1 M PB. Sections were treated with 0.2 mg/ml pepsin in 0.2 M HCl at 37 °C for 10-15 minutes and were washed in 0.1 M PB. Sections

were blocked in normal goat serum (NGS, 10%) made up in Tris-buffered saline (TBS, pH =7.4), followed by incubations in rabbit polyclonal anti-mGluR2/3 (1:1000; Chemicon, Temecula, CA) and mouse monoclonal anti-Cx36 (1:1000; Chemicon) primary antibodies diluted in TBS containing 2% NGS and 0.1% Triton X-100. Following several washes in TBS, the following secondary antibodies were used to visualize the immunoreactions: Alexa488 conjugated goat anti-rabbit and goat anti-mouse (1:500; Molecular Probes, Leiden, The Netherlands), Cy3 conjugated goat anti-mouse (1:500, Jackson ImmunoResearch, West Grove, PA). Sections were then mounted on slides in Vectashield (Vector Laboratories, Burlingame, CA). Images were taken with a confocal laser scanning microscope (FV1000, Olympus Europe) using a 20X (NA= 0.75) or a 60X (NA=1.35) objective. Automated sequential acquisition of multiple channels was used. Z-stack images were collected (at 0.5 – 2 µm Z directional steps). Either single confocal images or maximum intensity Z-projection images (6-15 images) are presented. For preembedding immunohistochemistry twenty three day-old C57BL6 mice were anesthetized and perfusion fixed first with a fixative containing 2% paraformaldehyde and 0.5% glutaraldehyde in 0.1M Na-acetate buffer (pH 6) for 2 minutes, followed by a fixative containing 2% paraformaldehyde and 0.5% glutaraldehyde in 0.1M Borate buffer (pH 8.5) for 30 minutes. The immunogold reactions were carried out as described in (Lorincz et al., 2002) using an anti-mGluR2/3 antibody (1:250; Chemicon, Temecula, CA).

Golgi cell model and network simulations

The model of the GoC pair and the GoC network were build using neuroConstruct (Neuroconstuct.org; (Gleeson et al., 2007)) and simulations were performed with NEURON (Carnevale and Hines, 2006) running on a standard desktop computer (pair), on a multicore Dell 9600, the SilverLab 240 core Lenovo cluster (network) or LEGION, the UCL supercomputer (network). For the network model a different GoC reconstruction than the ones for the 2-cell model in Fig.6 was used because it had a more complete axon, which contributed ~30% to the input conductance. Active conductances were implemented as previously described (Solinas et al., 2007). In short, the GoC model contains 2 types of Ca^{2+} currents with high and low activation threshold ($I_{Ca\text{-}HVA}$, $I_{Ca\text{-}LVA}$); 2 Ca^{2+} buffer mechanisms; two types of h-current (I_{HCN1}, I_{HCN2}); 3 voltage dependent K^+ currents (I_{KA}, I_{Kslow} (M-type), I_{Kv}), a mixed voltage and Ca^{2+} dependent K^+ current (I_{KC}) and a purely Ca^{2+} dependent K^+ current (I_{KAHP}, SK-type), and 3 types of Na^+ current (a transient I_{NaT}, a persistent I_{NaP} and a resurgent I_{NaR}). The specific membrane

resistance was 47.6 kΩ.cm^2, the specific axial resistance 100 Ω.cm and the specific membrane capacitance 1 μF.cm^{-2}. As in the original publication, all active conductances were placed in the soma without modification. The conductance waveform of the AMPA receptor type MF synapses (Kanichay and Silver, 2008) was based on measured EPSCs and had a single exponential rise (τ_0 = 0.1 ms) and a dual exponential decay (τ_1 = 0.7 ms, A_1 = 0.7 nS, τ_2 = 2.5 ms, A_2 = 0.2 nS, $E_{reversal}$ = 0 mV). The PF input conductance waveform had a single exponential rise (τ_0 = 0.1 ms) and a single exponential decay (τ_1 = 1.06 ms, A_1 = 0.67 nS, $E_{reversal}$ = 0 mV) (Dieudonne, 1998). Gaussian noise was added to the GoC pair model to reproduce the interspike interval variability observed in vitro (Solinas et al., 2007). In the network model, background synaptic noise was implemented with random trains of 20 MF synapses at 2 Hz each and 100 PF synapses at 0.5 Hz each. The somatic leak conductance for each cell was drawn from a uniform distribution (0 to 26 x 10^{-5} S.cm^{-2}; $E_{reversal}$, -55 mV) to increase the heterogeneity in input resistance and intrinsic firing frequencies across the GoC population. Spontaneous firing rate of individual cells in the network varied between 2 and 9 Hz with the majority firing between 6 and 9 Hz.

The thickness of the GCL modeled was determined experimentally by averaging measurements from the furrows to the apex of lobule 4 & 5Cb (80.6 μm, n=3). The footprint of the cuboid modeled (350 μm x 350 μm) was based on 2 considerations. 1) The number of electrical connections per GoC was not significantly different from a 600x600 μm network (Fig.S6B), and 2) Simulations with the larger network produced qualitatively similar results. GoCs were randomly distributed within the 350x35x80 μm rectangular box at a measured density of 4607 ± 166 cells/mm^3. Whether any 2 Golgi cells in the network model were electrically coupled was determined based on the radial distance between their somata. Thus first the radial distance between the somata of a pair of cells within the model was determined. Second, whether a connection was made was determined according to the experimentally obtained coupling probability function (P_c, see the Boltzmann function in Fig.7A, blue trace). This was implemented in Neuroconstruct with a Heaviside function (equation (1)). The Heaviside function takes as argument the subtraction of the probability function (P_c) with a random number between 0 and 1.

$$H[P_c - random(1)] \quad (1)$$

$$H[n] = 0 \; for \; n < 0, \; or \; 1 \; for \; n \geq 0$$

If the Heaviside function returned 1 the two cells made a connection. Finally, when a GoC pair had an electrical connection the conductance of the electrical synapse was determined based on the radial distance between their somata. We did this as follows; In Fig.7B we experimentally determined the relation between coupling coefficient (CC) and radial distance between GoC somata (exponential function, blue). However, this does not give us directly the relation between CC and the conductance of the electrical synapse. To determine this empirically, we used the 2-cell model (using the reconstructed cell for the network modeling). An electrical synapse was randomly positioned between the 2 cells on the dendrites, the electrical synapse conductance varied from 0 to 5 nS and the CC determined. This was repeated for 20 randomly positioned electrical synapses and averaged (Fig.S6A). This relation between coupling conductance (nS) and coupling coefficient (%) was fit with a dual exponential function; Coupling Conductance = 0.576 * exp(CC / 12.4) + 0.000590 * exp(CC / 2.79) – 0.564. This equation in conjunction with the equation for the spatial dependence of CC (exponential fit, Fig.7B, blue) gives the relation between the coupling conductance of the electrical synapse and the radial distance between GoC somata. We checked that randomly chosen coupled cells in the network gave coupling coefficients that matched well to the data in Fig.7B. For the network model only 1 GJ contact was made between each connected pair.

To model synchronous MF input in the GoC pair model, 20 randomly selected MF synapses were simultaneously activated (Fig.6I,J,K). However, for the network simulations (Fig.7, 8, 9) eight randomly selected MF synapses were activated per directly innervated cell. Each of these synapses became active after a random delay (uniform distribution) between 0 and 5 ms and remained activated at a mean rate of 200 Hz (Poisson distribution) for 10 ms. In addition, 50 randomly selected PF synapses per innervated cell were activated, 2 ms after the MF input started, each with a random delay between 0 and 5 ms. These PF synapses remained active for 15 ms at a mean rate of 350 Hz. In accordance with in vivo data, this pattern of input triggered predominantly spike doublets at ~100 to 250 Hz, and occasionally single or triplet spike responses. For every simulation in Fig.7E, F, Fig.8B, C, E, F and Fig.9A-D a different network

instantiation was used: network connections, the cells receiving the synaptic input, the activated synapses and synaptic delays were all randomly re-chosen. The time steps for simulating the coupled GoC pair and the network were 0.025 and 0.05 ms respectively. No difference was found when simulating with shorter time-steps.

4. Supplemental References

Barmack, N.H., and Yakhnitsa, V. (2008). Functions of interneurons in mouse cerebellum. J Neurosci *28*, 1140-1152.

Biro, A.A., Holderith, N.B., and Nusser, Z. (2005). Quantal size is independent of the release probability at hippocampal excitatory synapses. J Neurosci *25*, 223-232.

Carnevale, T., and Hines, M. (2006). The NEURON Book (Cambridge University Press).

Deans, M.R., Gibson, J.R., Sellitto, C., Connors, B.W., and Paul, D.L. (2001). Synchronous activity of inhibitory networks in neocortex requires electrical synapses containing connexin36. Neuron *31*, 477-485.

Dieudonne, S. (1998). Submillisecond kinetics and low efficacy of parallel fibre-Golgi cell synaptic currents in the rat cerebellum. J.Physiol *510 (Pt 3)*, 845-866.

Dugue, G.P., Brunel, N., Hakim, V., Schwartz, E., Chat, M., Levesque, M., Courtemanche, R., Lena, C., and Dieudonne, S. (2009). Electrical coupling mediates tunable low-frequency oscillations and resonance in the cerebellar Golgi cell network. Neuron *61*, 126-139.

Gleeson, P., Steuber, V., and Silver, R.A. (2007). neuroConstruct: a tool for modeling networks of neurons in 3D space. Neuron *54*, 219-235.

Golding, N.L., Mickus, T.J., Katz, Y., Kath, W.L., and Spruston, N. (2005). Factors mediating powerful voltage attenuation along CA1 pyramidal neuron dendrites. J.Physiol *568*, 69-82.

Kanichay, R.T., and Silver, R.A. (2008). Synaptic and cellular properties of the feedforward inhibitory circuit within the input layer of the cerebellar cortex. J.Neurosci. *28*, 8955-8967.

Lorincz, A., Notomi, T., Tamas, G., Shigemoto, R., and Nusser, Z. (2002). Polarized and compartment-dependent distribution of HCN1 in pyramidal cell dendrites. Nat.Neurosci. *5*, 1185-1193.

Ros, H., Sachdev, R.N., Yu, Y., Sestan, N., and McCormick, D.A. (2009). Neocortical networks entrain neuronal circuits in cerebellar cortex. J Neurosci *29*, 10309-10320.

Solinas, S., Forti, L., Cesana, E., Mapelli, J., De Schutter, E., and D'Angelo, E. (2007). Computational reconstruction of pacemaking and intrinsic electroresponsiveness in cerebellar Golgi cells. Front Cell Neurosci. *1*, 2.

Tamas, G., Buhl, E.H., Lorincz, A., and Somogyi, P. (2000). Proximally targeted GABAergic synapses and gap junctions synchronize cortical interneurons. Nat.Neurosci. *3*, 366-371.
